# Characterization of Commercial Metal Oxide Nanomaterials: Crystalline Phase, Particle Size and Specific Surface Area

**DOI:** 10.3390/nano10091812

**Published:** 2020-09-11

**Authors:** Michael Bushell, Suzanne Beauchemin, Filip Kunc, David Gardner, Jeffrey Ovens, Floyd Toll, David Kennedy, Kathy Nguyen, Djordje Vladisavljevic, Pat E. Rasmussen, Linda J. Johnston

**Affiliations:** 1 Metrology Research Centre, National Research Council Canada, Ottawa, ON K1A 0R6, Canada; MikeBushell@cmail.carleton.ca (M.B.); filipo.kunc@gmail.com (F.K.); David.Kennedy@nrc-cnrc.gc.ca (D.K.); 2 Health Canada, Environmental Health Research Science Bureau, 251 Sir Frederick Banting Driveway, Ottawa, ON K1A 0K9, Canada; pat.rasmussen@canada.ca; 3 X-ray Core Facility, University of Ottawa, STEM Complex, 150 Louis Pasteur, Ottawa, ON K1N 6N5, Canada; Dave.Gardner@uottawa.ca (D.G.); Jeffrey.Ovens@uOttawa.ca (J.O.); 4 Energy Mining & Environment Research Centre, National Research Council Canada, Ottawa, ON K1A 0R6, Canada; Floyd.Toll@nrc-cnrc.gc.ca; 5 Health Canada, New Substances Assessment Control Bureau, 269 Laurier Avenue West, Ottawa, ON K1A 0K9, Canada; kathy.nguyen@canada.ca (K.N.); djordje.vladisavljevic@canada.ca (D.V.)

**Keywords:** metal oxide nanomaterials, X-ray diffraction, transmission electron microscopy, specific surface area, crystalline phase

## Abstract

Physical chemical characterization of nanomaterials is critical to assessing quality control during production, evaluating the impact of material properties on human health and the environment, and developing regulatory frameworks for their use. We have investigated a set of 29 nanomaterials from four metal oxide families (aluminum, copper, titanium and zinc) with a focus on the measurands that are important for the basic characterization of dry nanomaterials and the determination of the dose metrics for nanotoxicology. These include crystalline phase and crystallite size, measured by powder X-ray diffraction, particle shape and size distributions from transmission electron microscopy, and specific surface area, measured by gas adsorption. The results are compared to the nominal data provided by the manufacturer, where available. While the crystalline phase data are generally reliable, data on minor components that may impact toxicity is often lacking. The crystal and particle size data highlight the issues in obtaining size measurements of materials with broad size distributions and significant levels of aggregation, and indicate that reliance on nominal values provided by the manufacturer is frequently inadequate for toxicological studies aimed at identifying differences between nanoforms. The data will be used for the development of models and strategies for grouping and read-across to support regulatory human health and environmental assessments of metal oxide nanomaterials.

## 1. Introduction

The physical chemical characterization of nanomaterials (NM) is an important prerequisite for studies aimed at assessing potential environmental and health effects. It has been shown that the unique physical and chemical properties of nanomaterials may influence their toxicity and behavior in the environment. Thus, data on these properties may inform potential NM risks. In addition, it is recognized that the lack of adequate characterization makes it impossible to compare data between labs and from different types of materials in many published studies [1], thus significantly limiting their value. This problem is exacerbated by the fact that nanomaterials are more complex than their corresponding bulk analogues and it may be necessary to evaluate a significant number of properties, including for example, chemical composition, particle shape and size, surface chemistry, crystal phase, dissolution and agglomeration. The measurement of some properties requires the dispersion of the material and reproducible dispersion protocols are either lacking or inconsistently applied [2]. The agglomeration/aggregation level of the material may affect its bioavailability, which is an important factor for risk assessment. Recent work is starting to address these data gaps by providing guidance on appropriate methods and best practices to measure various nanomaterial properties, and a general consensus as to the properties that must be assessed is emerging [3,4].

Many of the detailed studies on physical chemical properties of nanomaterials have focused on certified reference materials and representative test materials, such as those available as part of the European Commission Joint Research Centre Nanomaterial Repository. These studies provide important information on the level of agreement between laboratories and methods for standard materials, which are typically spherical, monodisperse and readily dispersible (for example, polystyrene, silica and gold nanoparticles) [5,6,7]. A recent study has aimed to address batch-to-batch variability in nanomaterials by synthesizing multiple batches of four different nanomaterials in different laboratories using several synthetic methods [8]. Eight different physical chemical properties were assessed for each material, providing valuable insight into the level of reproducibility for different batches prepared under carefully controlled conditions and the factors that lead to variability in reported data. Another study has started to address the issues that may be encountered with complex “real-world” materials. A number of industrial nanomaterials with different shapes and broad size distributions were characterized with multiple particle sizing methods and were compared to reference materials [9]. This study indicated that different methods could vary by up to a factor of five or more in the median particle size, with larger differences observed for commercial materials, as compared to standard reference materials.

Regulators face the challenge of developing a framework for commercial nanomaterials that are available from a number of suppliers and will have different physical chemical properties that are a function of the synthetic method, size and shape, and possibly other factors, such as storage conditions. The challenge is complicated by the observations that the measured properties of commercial materials do not always agree with the general specifications provided by the manufacturer. For example, a study from one of our groups has recently shown the importance of the mineral phase and particle size in determining the solubility of representative metal oxide nanomaterials [10]. As part of this study, X-ray diffraction (XRD) analysis demonstrated that some of the tested materials did not meet the manufacturer’s claims of the mineral phase [11]. Other work from our team has shown that commercial silica and zinc oxide nanomaterials vary significantly in the surface loading of functional groups, even for different batches obtained from the same supplier [12,13,14]. These studies highlight the need to better characterize the physical, chemical properties of commercial nanomaterials prior to using them for either application development or nanotoxicology studies.

In this work, we have undertaken the detailed characterization of a set of 29 commercial nanomaterials comprised of different nanoforms of aluminum, copper, titanium and zinc oxides. These materials have been identified by Health Canada as existing priority nanomaterials, which are currently in commerce in Canada, and data on physical chemical characterization and toxicological properties of these nanomaterials are required to fill the data needs for their regulatory risk assessments. While a number of previous investigations have characterized metal oxides used for nanotoxicology studies, most have only used one or two materials from a specific metal oxide family and the use of in-house synthesized materials or representative test materials is typical [15,16,17,18]. By contrast to previous studies, the nanomaterials we selected cover a range of commercial suppliers, particle sizes and surface treatments. The different nanoforms are anticipated to show different toxicological profiles. We have focused on several measurands that are important for basic characterization of dry nanomaterials and for determining dose metrics for nanotoxicology. We have examined the mineral phase (both crystalline and amorphous) using powder XRD, estimated the average crystallite diameter based on the diffraction peak broadening, measured particle shape and particle size distributions (as equivalent spherical diameters) by transmission electron microscopy (TEM) and estimated the specific surface area by the Brunauer-Emmett Teller (BET) method of gas adsorption. The results obtained are compared to the nominal values provided by the manufacturer. This provides insight into the reliability of the data provided by the manufacturer and the issues that are likely to be encountered in characterizing commercial materials. The work also provides a comprehensive data set from which the comparability of several methods for obtaining data that are relevant to dose metrics for nanotoxicological studies can be assessed.

## 2. Materials and Methods

### 2.1. Materials

A set of 29 nanomaterials was purchased from nine different suppliers: Sigma-Aldrich, Oakville, ON, Canada; Skyspring Nanomaterials Inc., Houston, TX, USA; Plasmachem GmbH, Berlin, Germany; NIST, Gaithersburg, MD, USA; Brenntag Canada, Toronto, ON, Canada; mKNano, Mississauga, ON, Canada; Nanostructured & Amorphous Materials Inc., Katy, TX, USA; US Research Nanomaterials Inc., Houston, TX, USA; BASF, Mississauga, ON, Canada. The materials consisted of four families of metal oxides: aluminum oxide, copper oxide, titanium oxide and zinc oxide. Various sizes, shapes, crystalline and amorphous phases and surface coatings were examined for each metal oxide. Details on the CAS number of the four metal oxides, and the sample code, supplier, purity and presence of coating for each nanoform are provided in Table 1.

### 2.2. X-ray Diffraction

Powder X-ray diffraction (XRD) patterns were obtained with a Rigaku Ultima IV powder diffractometer (Rigaku Americas Inc., The Woodlands, TX, USA) using monochromatic Cu Kα radiation (λ = 1.5406 Å) at 40 kV, 44 mA, a step scan size of 0.030° and a scan speed of 1°/min between 20 and 80° (2θ). The Ultima IV was configured for focused beam Bragg-Brentano (BB) with a monochromator. Powder samples were mounted in circular sample plates (2 mm deep, 24 mm diameter) and packed using a glass plate. Mineral phase identification was carried out using Rigaku analytical software (PDXL2 version 2.8), coupled with the Inorganic Crystal Structure Database (ICSD) and the International Centre for Diffraction Data (ICDD, PDF-2 Release 2015). Rietveld refinements and quantification were carried out using High Score Plus (Malvern Panalytical Inc., Westborough, MA, USA).

Various models have been developed to estimate the crystallite size from XRD profiles such as the Scherrer equation, Williamson-Hall plot, Size-Strain Plot, and the Halder-Wagner method [19]. In the present work, the average crystallite size for each analyzed nanomaterial was determined using the Williamson-Hall (W-H) plot model [20], as calculated using PDXL2 software. This model uses diffraction peak broadening from multiple diffraction peaks (i.e., at least four peaks), as a basis for estimating crystallite size, and includes a separate component for estimating peak broadening associated with crystallite microstrain. A spherical particle shape with cubic symmetry was the basis for interpreting crystallite size from diffraction data using this method. The LaB_6_ NIST standard (SRM 660c) was analyzed by the Rigaku Ultima IV to correct for the instrument contribution to peak broadening. The effective range for estimating crystalline size, using this method and diffractometer configuration, was from 3–100 nm [21,22]. Within this size range, the Williamson-Hall plot was well suited to determine crystallite size and the inherent microstrain within the nanoparticle of the microcrystalline structure [23,24,25].

### 2.3. Transmission Electron Microscopy

Samples were dispersed in deionized water by probe sonication using a 130 W ultrasonic processor (EW-04714-50, Cole-Parmer, Montreal, QC, Canada) equipped with a ¼ inch tip probe (EW-04712-14 Cole-Parmer) and operated at 50% amplitude with 30 s on/off cycles. Samples were dispersed at concentrations of 19 mg/mL for TiO_2_ and 2 mg/mL for other metal oxides and were immersed in an ice-water bath during sonication. Zeta potential of the suspensions was first measured in bent capillary dynamic light scattering (DLS) cuvettes as a function of pH using the Zetasizer Nano ZS (red) (Malvern Panalytical Inc.) equipped with a 632.8 nm HeNe laser and signal detection at 173°. Samples were then dispersed at the pH value at which the zeta potential had leveled off to a maximum, representing the highest surface charge. For TiO_2_ and Al_2_O_3_, this was typically pH < 3; for ZnO, the pH was not adjusted since the maximal charge was observed in deionized water. Samples with hydrophobic coatings such as caprylsilane and stearic acid were dispersed in ethanol. For all the samples, the DLS measurements as a function of the applied sonication energy were used to determine the point at which the size and/or polydispersity index had reached a plateau.

Dispersions sonicated with the optimal energy were diluted prior to depositing on formvar TEM grids (Ted Pella, Redding, CA, USA; Carbon Type-B on 200 mesh copper) that were treated with a 25/75 O_2_/Ar plasma for 1 min at 100 W. Two samples (100 µg/mL, 10 µg/mL) were prepared for each material and imaged using an FEI Tecnai G2 Spirit Twin TEM (ThermoFisher, Hillsboro, OR, USA) operated at 120 kV. The sample with the least aggregation and the larger number of individual particles was selected for analysis. Note that there was not a clear trend as to whether the higher or lower concentration sample gave the best results for TEM. Images were analyzed with ImageJ (1.52i, National Institutes of Health, Bethesda, MD, USA) using the manual polygon outlining tool to trace individual particles. Most samples showed a high degree of agglomeration/aggregation and many had areas of contamination. All identifiable particles in each image were analyzed, including touching particles as long as their boundaries could be clearly determined. Measurements of particle area, perimeter, minFeret and Feret were saved for each particle. The area was used to calculate an equivalent spherical diameter (d_eq_) and the Feret and minFeret to calculate the aspect ratio. Plots of size distributions and descriptive statistics were done in OriginPro 2019 (Origin Lab Corp, MA, USA).

### 2.4. Specific Surface Area

The BET method with nitrogen adsorption was used for the measurement of specific surface areas with an ASAP 2020 system from Micromeritics, Norcross, GA, USA. The cell containing the sample was weighed before degassing. The samples were heated at 10 °C/min to 110 °C, held for 10 min and then heated to 200 °C at 10 °C/min and held for 2 h. Specific surface area was determined by the multipoint BET method. Exceptions to this procedure were for Al-07 and Ti-011, which were degassed/heated at 10 °C/min to 90 °C, held for 10 min and then heated to 350 °C and held for 240 min.

## 3. Results

### 3.1. Crystalline Phase and Crystallite Size

The following subsections compare the expected crystal phases for each metal oxide explored in this study with the experimentally determined crystal phases and crystallite sizes. Powdered XRD patterns for each group of nanomaterials (i.e., Al_2_O_3_, CuO, TiO_2_, ZnO) are presented as stacked graphs in Figure 1 and Figure 2. Table 2 includes a comparison of analytical results for phase identification and crystallite size, against reported supplier information for phase identification and particle size. Detailed XRD patterns for each studied nanopowder, along with relevant reference compounds from the crystallographic databases, are included in the Supplementary Material (Figures S1–S29).

#### 3.1.1. Aluminum Oxides

Aluminum oxide (Al_2_O_3_) is commonly synthesized via the dehydration (calcination) of the Al hydroxide; the most stable form, *α*-Al_2_O_3_ (corundum), forms at around 1200 °C [26]. Transition, metastable phases occur at lower temperatures. The sequence *γ*-Al_2_O_3_→*δ*-Al_2_O_3_→θ-Al_2_O_3_→*α*-Al_2_O_3_ is observed as the temperature increases from 500 to 1200 °C. In contrast to the crystalline *α*-Al_2_O_3_, all transition phases have very low crystallinity [26,27].

**Figure 1:**
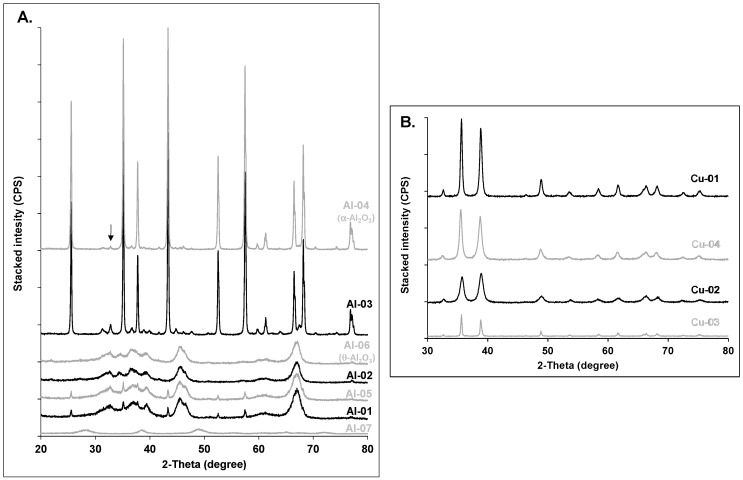
Stacked XRD patterns for (**A**) the aluminum oxides; Al-04 is representative of corundum (α-Al_2_O_3_; the arrow indicates traces of θ-Al_2_O_3_); Al-06 is representative of amorphous theta-phase (θ-Al_2_O_3_); (**B**) the copper oxides; all samples are 100% CuO. All fitting results are summarized in Table 2.

The crystalline *α*-Al_2_O_3_ dominated only in the Al-03 and Al-04 samples, in agreement with the phase characterization provided by suppliers, but contained a minor amount of θ-Al_2_O_3_. Samples Al-01, -02, -05, -06 and -07 were amorphous (Figure 1A, Table 2); Al-01 and Al-05 samples were contaminated with traces of the crystalline *α*-phase. The proportion of the latter could not be reliably modeled due to the low quality of the patterns. Based on the visual inspection of the XRD patterns, Al-01 and Al-05 exhibited similar patterns and Al-02 and Al-06 had comparable XRD signatures. In contrast to Al-02, however, the refined analysis of Al-06 using longer time scanning (0.3 deg/min) confirmed theta as the only phase (SI—Figure S6), in line with the supplier’s claim (Table 2). In agreement with the suppliers’ claim, the gamma-phase dominated in Al-01 and Al-05 (Figure 1A; Table 2).

The amorphous pattern of Al-07 was distinctly different from the previously described samples, as it was the only alumina sample in the form of nanowires (Figure 1A; Table 2). The average crystalline size (2 ± 1.2 nm) for the major mineral phase (dialuminum trioxide, Figure S7) was less than the particle diameter (5 nm) reported by the manufacturer.

The average estimated crystallite size of the amorphous phases in samples Al-01, -02, -05, -06 and -07 were generally smaller (<10 nm) than the particle size provided by the suppliers (Table 2). However, the average estimated crystallite size of 77 nm for the corundum in Al-04 greatly exceeded the suppliers’ claim of 40 nm (Table 2).

#### 3.1.2. Copper Oxides

In agreement with the suppliers’ characterization, all four tested powders were CuO and had mean crystallite sizes < 50 nm (Figure 1B, Table 2). Cu-03 had a mean crystallite size of 46 ± 15.3 nm and was within the standard deviation of the 40 nm size claimed by the supplier (Table 2). In contrast, the mean crystallite size of Cu-04 (19 ± 6.5 nm) was approximately 50% smaller than the particle size reported by the supplier. The Cu-02 crystallite size (14 ± 5.4 nm) was in good agreement with the nanotube diameters (10−12 nm) reported by the manufacturer. Note that for two of the nanowire/nanotube samples in this study (Al-07 and Cu-02), the Williamson-Hall plot provides a good estimate of crystallite thickness, but provides no information about length. Uvarov and Popov reported similar observations for nanorods and nanowires, in their study of X-ray diffraction techniques to determine crystallite size in 183 nanopowders [22].

#### 3.1.3. Titanium Oxides

TiO_2_ exists in the form of *β*-TiO_2_ (anatase) and *α*-TiO_2_ (rutile). Rutile is the most stable phase and forms at high temperature (1100 °C; [28]). The anatase occurs at lower temperatures and remains phase-pure over the range of 300 °C to 800 °C [28].

**Figure 2:**
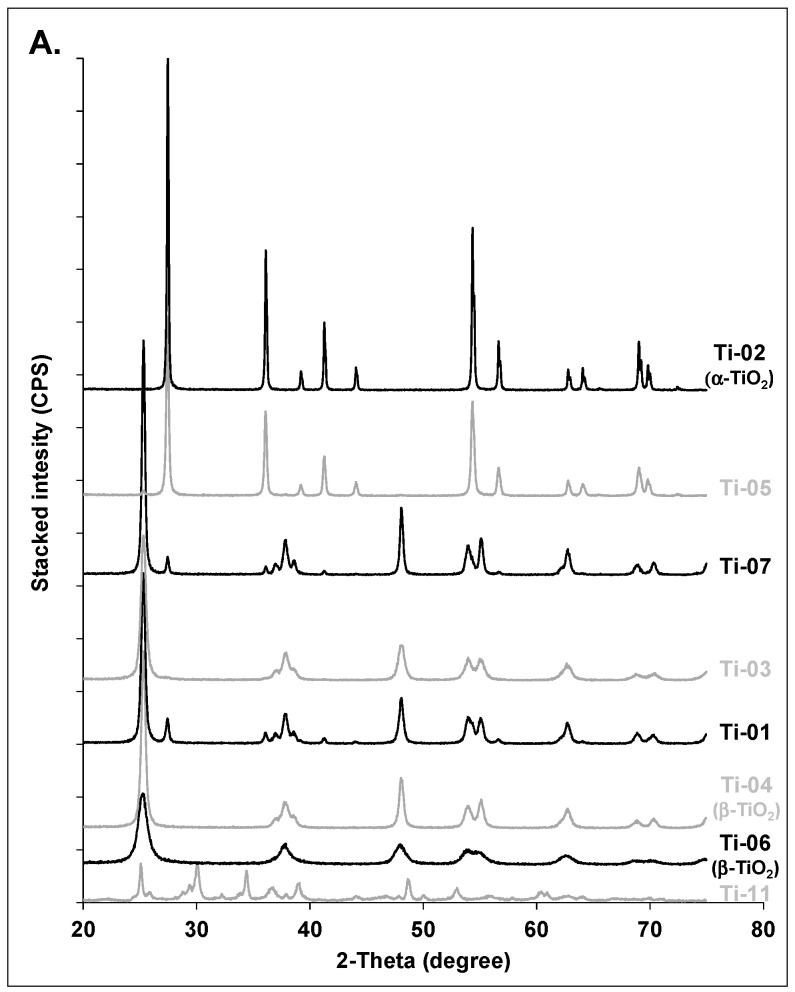

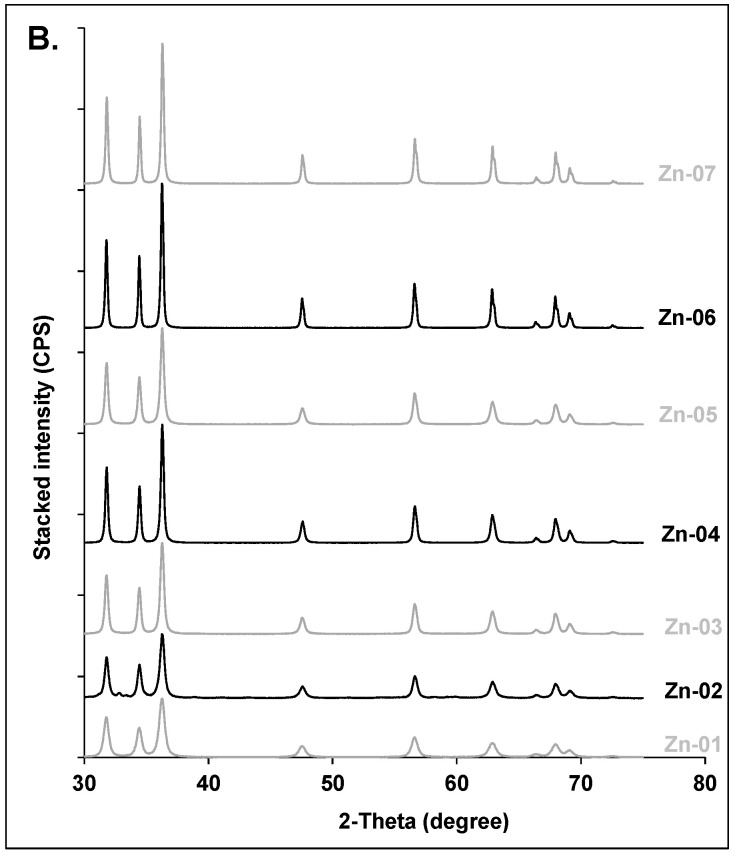
Stacked XRD patterns for (**A**) selected titanium oxides: Ti-02 is representative of 100% rutile (*α*-TiO_2_) while Ti-04 and Ti-06 are 100% anatase (*β*-TiO_2_); in-between are mixed species; Ti-08, -09, -10 exhibited similar XRD patterns to Ti-07 and are not shown. (**B**) zinc oxides; all samples are 100% ZnO. All fitting results are summarized in Table 2.

Most samples contained both phases in various proportions (quantitative estimates from Rietveld analysis), except for Ti-02 (100% rutile), Ti-04 and -06 (100% anatase; Figure 2A, Table 2). The identified phases were in good agreement with the characterization provided by the suppliers (Table 2). All the samples had crystallite sizes < 100 nm, except for Ti-02, which had an average crystallite size of 146 ± 23.7 nm. Compared to TEM particle size estimates of the diameter (Table 4, Ti-02 = 209 nm), the estimated average crystallite size for Ti-02 was approximately 30% smaller (particle size not reported by manufacturer). The crystallite sizes for Ti-03, Ti-04 and Ti-05 were less than the size claimed by the suppliers, especially for Ti-03 (16 nm vs. 50 nm) and Ti-04 (19 nm vs. 100 nm). The other nanopowders had crystallite sizes slightly larger than the particle size reported by the suppliers (Table 2). The average < 20 nm particle size claimed by Nanostructured & Amorphous Materials for the anatase/rutile mixtures Ti-07 to Ti-10 was generally exceeded in the tested lots: the estimated crystallite sizes were 30% to 45% larger than the claimed particle size for anatase (Table 2). In addition, the rutile component in Ti-08 and Ti-10 had a crystallite size of 49 and 46 nm respectively, indicating that overall the anatase/rutile mixture for these two powders did not meet the supplier’s claim. For Ti-11, anatase was likely present, but other detected phases could not be identified; there was a large discrepancy between the average crystallite estimates (25 ± 22.9 nm) and the nanowire diameter reported by the manufacturer (100 nm), suggesting that a polycrystalline nanowire was present.

**Figure 3:**
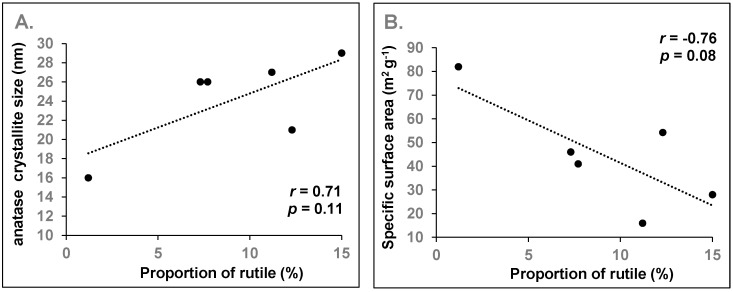
Correlation between (**A**) the average crystallite size of anatase and (**B**) the measured specific surface area of the nanopowder and the proportion of rutile in anatase/rutile mixtures with a low amount of rutile (≤15% of total TiO_2_); *r* is the Pearson’s correlation coefficient and *p* is the probability value.

For Ti-oxides obtained by thermal treatment such as calcination, Weibel et al. [28] reported the formation of pure-phase anatase below 800 °C, and noted that its crystallinity along with its crystallite size increased with increasing temperature. For example, they found that the nanoparticle size increased from 12 nm at 300 °C to 120 nm at 800 °C. Given that the pure phase of rutile formed at 1100 °C, a mixture of anatase and rutile would be expected between 800 °C and 1100 °C. In the study by Weibel et al. [28], the increased crystallinity was observable by a better resolution of the peaks at 2θ around 38 degrees in the XRD patterns. Likewise, this aspect is also observed in the present studied samples: the poorly crystalline Ti-06 anatase has only one broad peak at 2θ = 38°, as compared to the highly crystalline anatase samples (Ti-07 to 10), which exhibited several well defined peaks at the same position (Figure 2A). In mixed samples dominated by anatase, with a low amount of rutile (e.g., ≤15% of total TiO_2_; samples Ti-01, -03, -07, -08, -09, -10), the occurrence of rutile could thus be indicative of higher temperature treatment, and indirectly, of higher anatase crystallinity (and crystallite size). In fact, the average crystallite size of anatase tended to correlate with the proportion of rutile in these mixtures (*r* = 0.71; *p* = 0.11; Figure 3A).

#### 3.1.4. Zinc Oxides

In agreement with the expected composition, XRD identified pure-phase zincite (ZnO) in all samples (Figure 2B). All samples had average crystallite sizes ≤ 50 nm (Table 2). Sample Zn-05 had an estimated crystallite size 36% larger than the claimed particle size from the supplier (41 nm vs. 30 nm; Table 2).

### 3.2. Particle Shape and Particle Size Distribution

**Figure 4:**
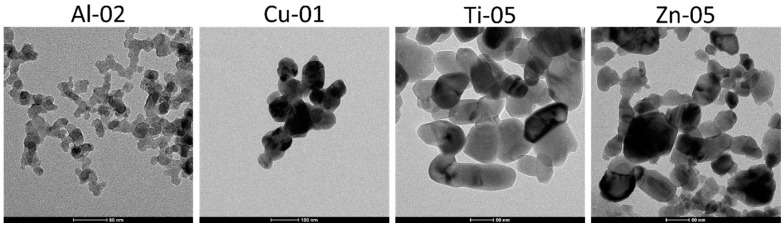
Representative TEM images for four metal oxides nanomaterials. Images for all samples are provided in the Supplementary Material.

TEM images were obtained at several resolutions for all the metal oxide nanomaterials. Representative images are shown in Figure 4 for one sample of each metal oxide and representative images for all samples are provided as Supplementary Material (Figures S30–S34). The TEM contrast was the highest for CuO samples, intermediate for ZnO and TiO_2_ and relatively low for most Al_2_O_3_ samples. The contrast was significantly better for Al-04 than for most other aluminum oxides, possibly due to either the larger particle size or the large fraction of crystalline α-Al_2_O_3_ for this sample. All the samples showed a relatively high degree of agglomeration/aggregation. However, it was possible to locate regions where individual particles could be identified and analyzed for most samples. Exceptions included Al-01, Al-03 and Ti-06, for which it was not possible to identify areas that were suitable for size analysis of individual particles. This was due to a combination of low contrast and heavily aggregated or small particles. The particle count was also low for two CuO samples, again due to strongly aggregated particles. Contamination was frequently observed for some samples (see the images for Zn-02, Ti-03 and Ti-11 in Figures S32 and S33) and may be due to a residual inorganic matrix from the material synthesis. In some cases, representative images of the samples were provided on the manufacturer’s website and were qualitatively similar to the data provided here.

The size and shape information provided by the manufacturer is provided in Table 3, along with particle size and shape data, as measured by TEM in the present study. The available manufacturer’s information on the particle shape indicated that most particles were either spherical or ellipsoidal. However, TEM images frequently indicated a range of shapes varying from approximately spherical/circular to irregular ellipsoidal or oval-shaped particles, squares, rectangles (often with rounded corners) and hexagons, as summarized in Table 3 and illustrated in Figures S30–S33. One CuO sample (Cu-02) was predominantly nanorods and was consistent with the supplier quoted nanotube shape, although other irregular shaped particles were also present. Two other samples (Al-05 and Zn-07) contained a population of nanorods that did not account for a large fraction of the particles. Samples Al-07 and Ti-11 were predominantly interconnected nanowires and nanorods, respectively, as expected from the supplier information. However, a single, reasonably uniform shape was not observed for any of the other samples, although the diversity of observed shapes varied from sample to sample.

**Figure 5:**
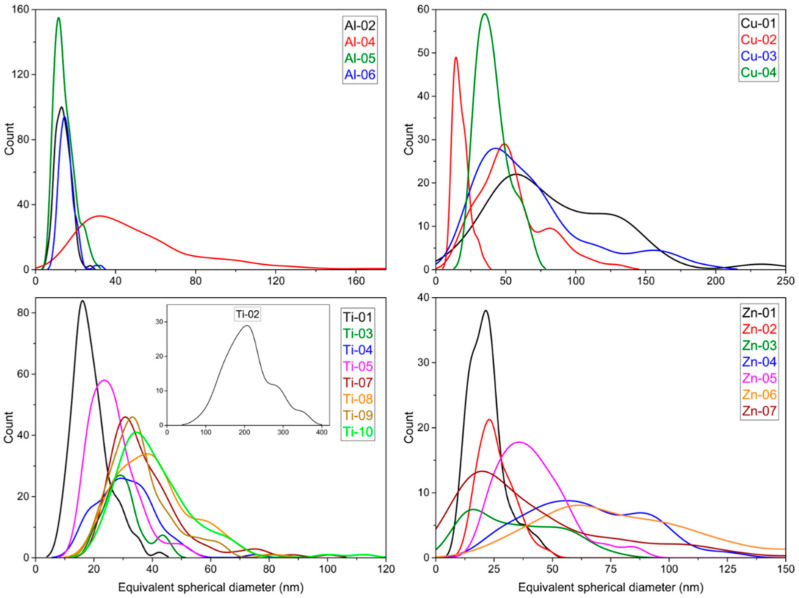
Smooth histogram plots illustrating the distributions for the equivalent spherical diameter for the various metal oxides. Two plots (Feret and minFeret in red) are shown for the nanorod sample, Cu-02. The insert in the bottom left plot shows the Ti-02 histogram on a larger *x*-axis scale.

The particle size distributions measured from TEM images are illustrated in Figure 5, which shows the kernel smooth distributions that are obtained from the histograms for the equivalent diameter, d_eq_, for each data set. Histograms for all the samples are provided in Figures S35–S38. The equivalent diameter is the diameter of a circle that has the same area as the particle; this approach is typically used for non-spherical and/or irregular shaped particles. The plots indicate that many of the particle size distributions are quite broad, indicating high polydispersity, and are typically asymmetric. Table 3 summarizes the particle size distributions as mean d_eq_ with standard deviation as a measure of the width of the particle size distribution for each data set. Note that this relatively simple metric does not adequately account for the observed asymmetric size distributions (Figure 5). In many cases, the standard deviations are similar in magnitude to the mean, which is indicative of high polydispersity. No attempt has been made to fit the data to a distribution, given the relatively limited statistics and the high degree of agglomeration/aggregation observed for most samples. The mean aspect ratio (based on the Feret/minFeret ratio) and standard deviation are also summarized in Table 3. Mean Feret and minFeret values are listed for four samples that have a significant population of rod-shaped particles. Note that only width was measured for Ti-11 since the nanowires were branched and interconnected making it difficult to measure their length.

**Figure 6:**
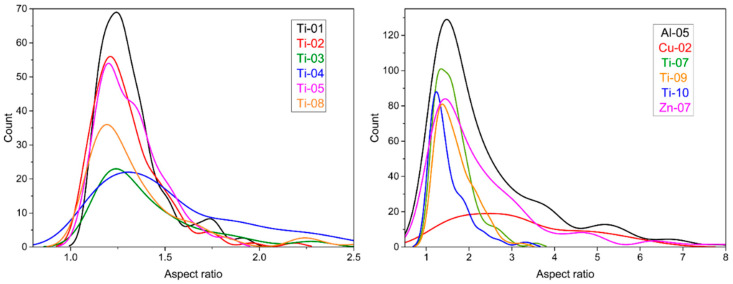
Smoothed histogram plots for aspect ratio for representative metal oxides nanomaterials. TiO_2_ samples with mean aspect ratios < 1.5 are shown in the left panel and several samples with higher mean aspect ratios are shown in the right panel.

The values for mean d_eq_ in Table 3 agree reasonably well with the supplier quoted size (presumably diameter although this is not explicitly stated in most cases) for some samples, but are significantly different for others. For example, measured values for Zn-02, Cu-04, Al-02 and Ti-03 are the same as the nominal value from the supplier. By contrast, other samples had mean values for d_eq_ that were as much as twice as large (Zn-04, Ti-08) or significantly smaller than (Ti-04) the manufacturer’s quoted value. Note that, with one exception, all the materials had mean sizes that were below 100 nm, which was still well within the nanoscale range. The exception was Ti-02, for which few particles (~3%) had d_eq_ < 100 nm; supplier’s information on particle size was not available for this sample. A further complication in comparing the TEM and supplier data was the relatively broad size distributions (as indicated by the standard deviations listed in Table 3) measured by TEM. This may mean that an insufficient number of particles were measured to provide reliable supplier data. While there are a range of particle shapes for the various samples, the aspect ratios cover a fairly narrow range with mean values generally between 1.1 and 1.5 and very few particles with aspect ratios above ~2 (Table 3). This is illustrated in Figure 6 (left) for the TiO_2_ samples. Exceptions were the nanotube and nanowire samples, Cu-02 and Ti-11, two samples with a significant fraction of nanorods, Al-05 and Zn-07, and two samples with higher aspect particles, Ti-07 and Ti-09 (Figure 6, right).

### 3.3. Comparison of Particle Size Measurements

**Figure 7:**
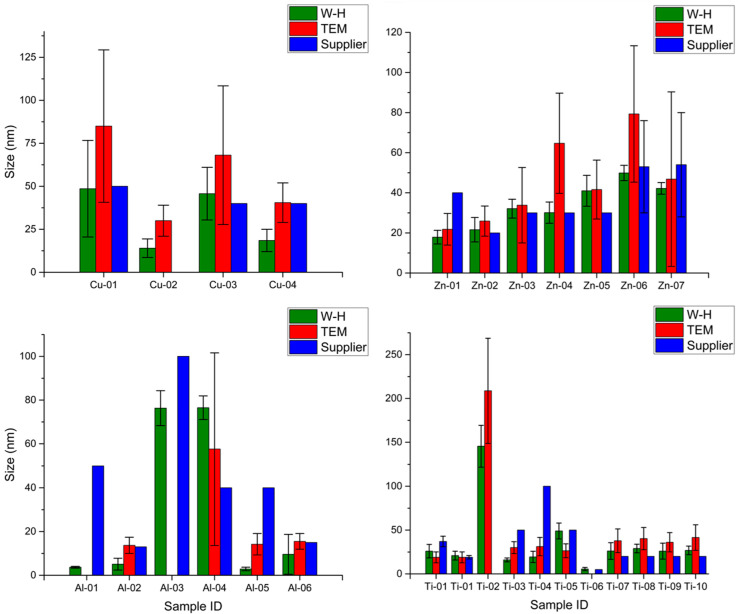
Comparison of nominal size reported by the supplier to the mean crystallite size from XRD and the TEM particle size distribution. The error bars for XRD are the standard deviation of the estimated size obtained from the Williamson-Hall linear regression, whereas for TEM they represent the standard deviation as a measure of the width of the particle size distribution. Nanowires (Al-07, Ti-11) are not included.

Figure 7 summarizes the particle sizes measured by TEM and XRD and the nominal size quoted by the supplier where available. This allows for a comparison of the agreement between the two methods used here and between our measured values and the data provided by the supplier. It should be noted that the size estimates from TEM and XRD may be different [15]. A particle can be composed of one or more crystallites and microscopy methods will generally result in larger particle size estimates than the crystallite size estimates calculated from the Williamson-Hall (W-H) analysis of XRD data. Excluding the three nanowire/nanotube samples, 21 of the 23 materials for which both TEM and XRD data are available have XRD sizes that are similar to or smaller than the TEM values. The exceptions are Al-04 and Ti-05 (Table 2 and Table 3), although it should be noted that Al-04 has a broad size distribution. The width for each of the three nanowire/nanotube samples measured as the TEM minFeret value is larger than the estimated nanowire diameter from the XRD measurements of crystallite size.

Information on the source of the nominal particle size quoted by the manufacturer was only available for some of the nanomaterials. Size information for Ti-01 was obtained from XRD measurements, and for approximately one third of the remaining samples, the manufacturer’s information indicates that nominal sizes are based on TEM data. However, it is not clear whether the values are mean sizes based on measuring a statistically relevant number of particles; this appears unlikely for the cases where approximate values or upper limits are provided. An additional consideration is the lack of information on whether the TEM was measured for never-dried or redispersed materials, which may behave differently. Comparing the three different values for the estimated particle size, there are four samples for which both TEM and XRD agree with the supplier value within ±25%, four where both TEM and XRD estimates are lower than the nominal value and six where both values are higher than the nominal value. For the remaining seven samples, one of our two measured size values (either XRD or TEM) was close to the nominal value and the other was either higher or lower. None of the discrepancies in size were larger than a factor of ~2 and in no cases did the mean sizes exceed 100 nm. The latter observation means that all materials could be classified as nanomaterials, even if the size range was not particularly well-defined. Note that supplier information was unavailable for Ti-02, which was the only sample for which TEM and XRD gave a particle size > 100 nm.

The above noted variations in size are not very surprising since these commercial nanomaterials are challenging samples for microscopy measurements given the high level of aggregation and irregular particle shapes and since XRD and TEM are anticipated to give different size estimates. Nevertheless, the lack of agreement between the measured data and the supplier’s nominal size estimates would make it challenging to extract any meaningful correlations with particle size for these samples. Consider Al-04 and Al-05 as an example. These materials have different crystalline phases but the same particle size based on the supplier information, indicating that one could use this pair to test for differences between crystalline and amorphous phases. However, both TEM and XRD data indicate that the nominal size is a significant underestimate for Al-04 and an overestimate (by at least a factor of three) for Al-05. Therefore, any differences between the two cannot be assigned to differences between the crystalline and amorphous phases. A similar situation arises for Ti-03 and Ti-04, which are confirmed to be predominantly anatase, as indicated by the supplier, and which have nominal sizes that differ by a factor of two. However, the measured TEM data indicates that the supplier-reported sizes are overestimates and the two materials do not have significantly different sizes. As a third example, the set of unmodified TiO_2_ and surface coated TiO_2_ (Ti-07 to Ti-10) from Nanostructured & Amorphous Materials all have a nominal size of 20 nm, but TEM and XRD values were higher by as much as a factor of two.

### 3.4. Specific Surface Area

Specific surface area (SSA) has been suggested as an appropriate alternative to mass or number based dose metrics [29]. SSA values for the various metal oxides were measured by the BET method using the adsorption of nitrogen and a summary of the data can be found in Table 4 with a comparison to the nominal values provided by the suppliers. The measured value for Ti-01, a NIST standard reference material, agrees well with the certified value and is within the range of values measured for this material as part of an interlaboratory comparison validation study [30]. For the other 19 samples for which the supplier provides data, the BET measured SSA values are within approximately 20% of this nominal value for 11 samples, but vary by a larger amount (up to five-fold) for the other eight samples. Some of the supplier data are provided as a range or a lower limit, which may indicate a nominal value based on multiple batches of the material. The discrepancies between the measured values and those provided by the supplier may well reflect batch-to-batch variability. Table 4 also provides SSA values for selected metal oxide nanomaterials that are estimated from the material density and the surface area calculated from the TEM size. There are only four materials for which the measured and calculated SSA values are the same at within ± 10%; for 14 materials the calculated SSA values are larger than the measured values, although rarely by more than a factor of two. The lower SSA values measured by BET are consistent with the formation of aggregated particles that have lower accessible surface area than was calculated from the mean size of the (individual, unaggregated) particles measured by TEM. The broad and asymmetric size distributions (Figure 5) may also contribute to the difference between measured and calculated SSA values.

**Figure 8:**
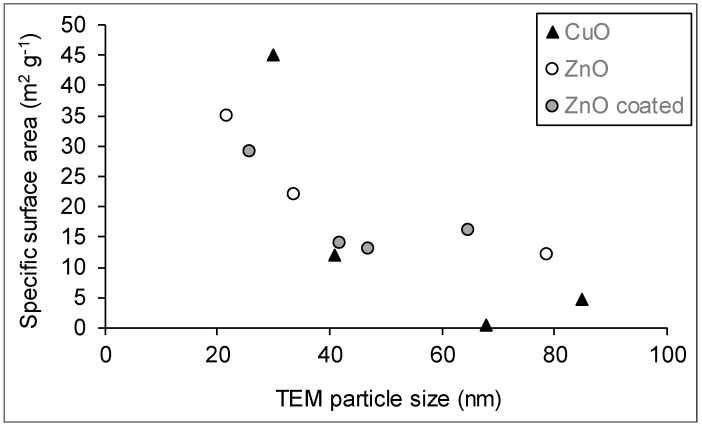
Relationships between the measured specific surface area and the particle size measured by TEM for CuO and ZnO nanomaterials: all CuO powders were uncoated; ZnO coated particles are described in Table 1.

Particle size, mineral phase and crystallinity are the main factors influencing the specific surface area of nanomaterials. There is no obvious correlation between SSA and particle size as a single factor. For example, Ti-03, Ti-04 and Ti-05 have comparable crystallite sizes from XRD measurements, but SSA values for Ti-03 and Ti-04 are four times greater than that for Ti-05 (Table 2, Table 3 and Table 4). This result is explained by the difference in mineral phases: Ti-05 is crystalline rutile whereas Ti-03 and Ti-04 are anatase minerals (Figure 2A). The combined effects of crystalline phase and particle size are also evident when comparing SSA for Al-04 (crystalline *α*-Al_2_O_3_) and Al-05 (amorphous *γ*-Al_2_O_3_). The estimation of the SSA from the mean TEM size and material density indicates that the SSA of Al-05 should be approximately six times that of Al-04, which is consistent with the trend in the measured BET values (Table 4). However, when comparing samples with similar mineral phases within a given group of metal oxides, SSA values generally tended to decrease with the increase in particle size for both CuO and ZnO powders, as expected (Figure 8). For ZnO, the SSA is estimated to decrease from 49 to 14 m^2^/g, with an increase in particle size from 22 nm (Zn-01) to 79 nm (Zn-06). The measured SSA values for most samples are lower than the estimates based on particle size, which may be due to particle aggregation, particularly for the samples with hydrophobic coatings or functional groups (e.g., Zn-04 and Zn-05). A comparable trend was observed for the mixed anatase samples. In a previous section, we demonstrated that an increase in the rutile fraction could be used as an indicator of higher anatase crystallinity (and associated crystallite size; Ti-01, -03, -07, -08, -09 and -10; Table 2; Figure 3A). Accordingly, the increase in the rutile fraction was correlated to a decrease in SSA in these samples (r = 0.76; *p* = 0.08; Figure 3B).

## 4. Implications for Risk Assessment

This study provides crystalline phase and crystallite size measurements by XRD, particle size and shape data from TEM and SSA data for 29 commercial metal oxide nanomaterials. These are important measurands for quality control purposes when these nanomaterials are used in applications and for assessments of their potential human health and environmental impacts. They are also key metrics for assessment of applied dose for nanotoxicological studies and for implementing read across strategies for regulatory purposes. The data presented indicate that the supplier information for crystalline phase is generally reliable, although measurable amounts of unreported additional phases are detected in some samples. Note that the crystalline phase has been shown to affect dissolution rates of nanomaterials, which may in turn explain observed nanotoxicological responses [31]. The effects of crystalline phase are particularly significant for TiO_2_, for which the surface area and crystalline phase (amorphous, rutile, anatase) correlate with the capacity to generate reactive oxygen species [32,33]. Finally, crystal structure is also important in considering the correct refractive index for dynamic light scattering and for the conversion of mass to particle number dose-metrics [10,34].

TEM and XRD estimated crystallite/particle sizes do not agree with the supplier data for a significant number of samples, which is an important consideration for any nanomaterials that show size-dependent cellular uptake or properties. Note that some variations between TEM and XRD are to be expected since some particles may contain multiple crystallites. Furthermore, the metal oxide nanomaterials studied here are challenging samples for size analysis, given their broad size distributions and high level of aggregation. Nevertheless the results highlight issues that are likely to be encountered in cases where reliable size information is necessary to ensure that the material properties are adequately documented for comparison with other materials and to test for the effects of physical chemical parameters on environmental and health impacts. This will be of crucial importance in developing read across approaches [35,36] to minimize the amount of physical chemical and toxicological data that is needed to develop regulatory guidelines for nanomaterials. These characterization data will allow Health Canada to fill data gaps for priority nanomaterials and to evaluate the relationships between characteristics of representative nanoforms and their toxicological properties for understanding the potential risks arising from the use of nanomaterials. Data obtained from this study will be useful for the development of models and strategies for grouping and read-across to support regulatory human health and environmental assessments of the nanomaterials in Canada.

## Figures and Tables

**Table 1:** List of the metal oxides studied, their supplier, reported purity and coating.

Code	Formula ^1^	Trade Name ^2^	Supplier ^3^	Purity	Coating
Al-01	Al_2_O_3_	N/A	Sigma Aldrich (cat no. 544833)		uncoated
Al-02	Al_2_O_3_	Aeroxide Alu C	Sigma Aldrich (cat no. 718475)	99.8%	uncoated
Al-03	Al_2_O_3_	N/A	Skyspring Nanomaterials, Inc.	99.99%	hydrophobic coating
Al-04	Al_2_O_3_	Aluminium oxide, alpha phase	Plasmachem GmbH	99.8%	uncoated
Al-05	Al_2_O_3_	Aluminium oxide, gamma phase	Plasmachem GmbH	>99.9%	uncoated
Al-06	Al_2_O_3_	Aluminium oxide, theta phase	Plasmachem GmbH	99.8%	uncoated
Al-07	Al_2_O_3_	N/A	mkNano	99%	unspecified
Cu-01	CuO	N/A	Sigma Aldrich (cat no. 544868)	>95%	uncoated
Cu-02	CuO	N/A	Sigma Aldrich (cat no. 792004)	100%	uncoated
Cu-03	CuO	N/A	mkNano	99%	uncoated
Cu-04	CuO	N/A	Plasmachem GmbH	>99%	uncoated
Ti-01	TiO_2_	SRM 1898/Aeroxide TiO_2_ P25	NIST	99.4%	uncoated
Ti-02	TiO_2_	Kronos 2310 titanium dioxide	Brenntag Canada	>92.5%	surface treated with Al, Si and Zr compounds
Ti-03	TiO_2_	TiO_2_-Anatase, 50 nm	mkNano	98%	uncoated
Ti-04	TiO_2_	TiO_2_-Anatase, 100 nm	mkNano	99.7%	uncoated
Ti-05	TiO_2_	TiO_2_-Rutile, 50 nm	mkNano	99%	uncoated
Ti-06	TiO_2_	TiO_2_-Anatase, 5 nm, water dispersible	mkNano	99.9%	uncoated
Ti-07	TiO_2_	Titanium oxide, silica coated anatase/rutile, 20 nm	Nanostructured & Amorphous Materials, Inc.	>96%	silica coated
Ti-08	TiO_2_	Titanium oxide, silica & alumina coated, anatase/rutile, 20 nm	Nanostructured & Amorphous Materials, Inc.	>92%	silica and alumina coated
Ti-09	TiO_2_	Titanium oxide, silica & stearic acid coated anatase/rutile, 20 nm	Nanostructured & Amorphous Materials, Inc.	>96%	silica & stearic acid coated
Ti-10	TiO_2_	Titanium oxide, silica & silicone oil coated anatase/rutile, 20 nm	Nanostructured & Amorphous Materials, Inc.	>92%	silica & silicone oil coated
Ti-11	TiO_2_	N/A	Sigma (cat no. 774510)		unspecified
Zn-01	ZnO	N/A	US Research Nanomaterials Inc.	>99%	uncoated
Zn-02	ZnO	N/A	Skyspring Nanomaterials, Inc. (Cat no. 8412DL)	99%	treated w/stearic acid
Zn-03	ZnO	N/A	Nanostructured & Amorphous Materials, Inc.	>99%	uncoated
Zn-04	ZnO	N/A	Nanostructured & Amorphous Materials, Inc.	98%	3-amino-propyltri-ethoxysilane
Zn-05	ZnO	N/A	Nanostructured & Amorphous Materials, Inc.	98%	silicone oil (lipophilic)
Zn-06	ZnO	Z-COTE	BASF	100%	uncoated, hydrophobic
Zn-07	ZnO	Z-COTE HP1	BASF	97.5%	triethoxy-caprylylsilane

^1^ Al_2_O_3_: CAS no. 1344-28-1; CuO: CAS no. 1317-38-0; TiO_2_: CAS no. 13463-67-7; ZnO: CAS no. 1314-13-2. ^2^ Particle sizes are provided to distinguish different nanoforms for some TiO_2_ samples; supplier size data for all the samples are provided in Table 2 and Table 3. ^3^ Supplier refers to the company from which the material was purchased. Al-02 and Ti-01 were sourced by the supplier from Evonik.

**Table 2:** Comparison of the XRD results with the phases and particle size provided by the suppliers for the various metal oxides.

	XRD Results			Suppliers	
Sample	Phases Detected	Crystallite Size, nm	±SD	Phases Detected	Particle Size, nm
Al-01	Gamma/delta Al_2_O_3_ *^1^*	4	0.4	gamma phase	< 50
	Minor: Corundum α-Al_2_O_3_)	53	9.6	N/A *^2^*	N/A
Al-02	Delta-Al_2_O_3_	5	2.7	N/A	13 *^3^*
	Traces corundum (α-Al_2_O_3_)	N/A	N/A	N/A	N/A
Al-03	Corundum (α-Al_2_O_3_)	76	8.0	alpha phase	<100
	Minor: Theta-Al_2_O_3_	11	2.4	N/A	N/A
Al-04	Corundum (α-Al_2_O_3_)	77	5.4	alpha phase	40
	Minor: Theta-Al_2_O_3_	13	3.5	N/A	N/A
Al-05	Gamma-Al_2_O_3_	3	0.8	gamma phase	40
	Minor: Corundum (α-Al_2_O_3_)	49	38.6	N/A	N/A
Al-06	Theta-Al_2_O_3_	10	9.1	theta phase	15
Al-07	Dialuminum trioxide	2	1.2	N/A (nanowires)	5 × 1000
	Minor: Aluminum oxide	10	2.5		
Cu-01	100% CuO	49	28.1	CuO	<50
Cu-02	100% CuO	14	5.4	CuO (nanotubes)	(10−12) × (75−100)
Cu-03	100% CuO	46	15.3	CuO	40
Cu-04	100% CuO	19	6.5	CuO	40
Ti-01	87.7% Anatase (β-TiO_2_)	21	5.0	Anatase, 76%	19 ± 2
	12.3% Rutile (α-TiO_2_)	26	7.7	Rutile, 24%	37 ± 6
Ti-02	100% Rutile (α-TiO_2_)	146	23.7	Rutile	
Ti-03	98.8% Anatase (β-TiO_2_)	16	2.2	Anatase	50
	1.2% Rutile (α-TiO_2_)	N/A	N/A		
Ti-04	100% Anatase (β-TiO_2_)	19	6.4	Anatase	100
Ti-05	98.8% Rutile (α-TiO_2_)	49	9.0	Rutile	50
	1.2% Anatase (β-TiO_2_)	27	10.9		
Ti-06	100% Anatase (β-TiO_2_)	6	1.8	Anatase	5
Ti-07	92.7% Anatase (β-TiO_2_)	26	9.5	Anatase, 80–90%	20
	7.3% Rutile (α-TiO_2_)	N/A	N/A	Rutile, 10–20%	
Ti-08	85% Anatase (β-TiO_2_)	29	4.9	Anatase, 80–90%	20
	15% Rutile (α-TiO_2_)	49	11.0	Rutile, 10–20%	
Ti-09	92.3% Anatase (β-TiO_2_)	26	9.1	Anatase, 80–90%	20
	7.7% Rutile (α-TiO_2_)	N/A	N/A	Rutile, 10–20%	
Ti-10	88.8% Anatase (β-TiO_2_)	27	4.9	Anatase, 80–90%	20
	11.2% Rutile (α-TiO_2_)	46	6.4	Rutile, 10–20%	
Ti-11	Beta-TiO_2_Other phases: unidentified	25	22.9	N/A (nanowire)	100 × 10,000
Zn-01	100% Zincite (ZnO)	18	3.4	ZnO	35–45
Zn-02	100% Zincite (ZnO)	22	6.1	ZnO	10–30
Zn-03	100% Zincite (ZnO)	32	4.7	ZnO	30
Zn-04	100% Zincite (ZnO)	30	5.3	ZnO	30
Zn-05	100% Zincite (ZnO)	41	7.7	ZnO	30
Zn-06	100% Zincite (ZnO)	50	3.8	ZnO	53 ± 23
Zn-07	100% Zincite (ZnO)	42	2.9	ZnO	54 ± 26

^*1*^ ForAl_2_O_3_ nanopowders: Quantitative estimates of crystalline vs. amorphous phases by Rietveld analysis could not be performed due to the low quality of patterns. The dominant phase is reported first, followed by minor phases if present (<2%). ^*2*^ Not available. ^*3*^ Particle size = 13 nm; aggregate size = 140 nm.

**Table 3:** Comparison of particle shape, particle size distribution (equivalent diameter, d_eq_ and aspect ratio) as determined experimentally by TEM with shape and size data provided by the suppliers.

Sample	TEM						Supplier *^1^*	
	Shape *^2^*	n *^3^*	d_eq_, (std Error), nm *^4^*	SD, nm *^4^*	Aspect Ratio	SD	Shape	Size, nm (Method)
Al-01	nanorods, IR	N/A						<50 (TEM)
Al-02	SP, EL, IR	128	13.8 (0.3)	4	1.4	0.2		13 *^5^* (TEM)
Al-03	EL, IR	N/A						<100
Al-04	~SP, HX	158	58 (3)	44	1.1	0.2	spherical	~40 (TEM)
Al-05	nanorods, plates	232	14.2 (0.3) *21, 10*	5	2.3	1.2	spherical, elongated	~40 (TEM)
Al-06	EL, IR	111	15.5 (0.4)	4	1.3	0.3		~15
Al-07	nanowires	119	*6.0 (0.1) ^6^*	*1.2*			nanowire	5 × 1000 *^6^*
Cu-01	SP, EL	78	85 (5)	44	1.3	0.2		< 50 (TEM)
Cu-02	nanorods, IR	71	30 (1) *54, 18*	9	3.1	1.4	nanotube	(10−12) × (75−100)
Cu-03	SP, IR	108	68 (4)	40	1.3	0.2		40
Cu-04	SP, EL, IR	105	41 (1)	12	1.4	0.2	spherical	~40
Ti-01	SP, EL, EL, RC	137	18.7 (0.5)	6	1.3	0.2		19, anatase; 37, rutile (XRD)
Ti-02	SP, EL, IR	129	209 (5)	60	1.3	0.2		
Ti-03	IR	52	30 (1)	7	1.4	0.3		50
Ti-04	EL, IR	75	31 (1)	10	1.5	0.4		100
Ti-05	EL, RC, IR	114	26.4 (0.7)	8	1.3	0.2		50
Ti-06	single particles not detected	N/A					<5	<5
Ti-07	EL, RC, IR	121	38 (1)	14	1.6	0.4	spherical, ellipsoidal	20 (TEM)
Ti-08	EL, RC, IR	100	40 (1)	13	1.4	0.3	spherical, ellipsoidal	20 (TEM)
Ti-09	SP, EL, RC	106	36 (1)	11	1.7	0.4	spherical	20
Ti-10	SP, EL, IR	101	42 (1)	15	1.5	0.5	spherical	20
Ti-11	Nanorods	90	*790 (40), 65 (3)*	*377, 32*	13	7	nanowires	100 × 10,000
Zn-01	SP, EL	132	22 (1)	8	1.4	0.2	nearly spherical	35–45 (TEM)
Zn-02	SP, EL, HX	77	26 (1)	8	1.3	0.2		10–30
Zn-03	SP, EL, RC	71	34 (2)	19	1.3	0.2	spherical	30 (TEM)
Zn-04	SP, EL, RC	124	65 (2)	25	1.3	0.2	spherical	30
Zn-05	SP, EL, IR	129	42 (1)	15	1.3	0.2	spherical	30
Zn-06	EL, IR	132	79 (3)	34	1.4	0.3		53 ± 23
Zn-07	rods, EL, RC	154	47 (3)	44	2.3	1.5		54 ± 26

*^1^* Size and shape are not provided for all samples. TEM and XRD are noted in parentheses if the product information indicates the method for size analysis. *^2^* Shapes abbreviated as: SP = (nearly) spherical; EL = ellipsoidal or oval; RC = rectangular; HX = hexagonal; IR = irregular, typically an irregular polygon. Nanorods and nanowires are distinguished based on whether they are straight (nanorods) or bent and/or branched (nanowires). *^3^* N/A indicates that the images were not analyzed because the aggregation or lack of contrast made it difficult to identify a sufficient number of individual particles. *^4^* Mean Feret and minFeret values are shown in italics for samples that have a significant population of nanorods. *^5^* Aggregate size of 140 nm. *^6^* Interconnected and branched nanowires for which only width was measured.

**Table 4:** Comparison of specific surface area measured by the BET method with supplier information and the approximate SSA values calculated from the TEM equivalent diameter.

Sample	Specific Surface Area, m^2^/g		
	BET	Supplier *^1^*	Calculated (TEM) *^2^*
Al-01	129	>40	
Al-02	97	85–115	
Al-03	16, 35 *^3^*		
Al-04	9.8	>10	25.9
Al-05	124	>40	
Al-06	100	90–110	
Al-07	466		
Cu-01	4.6	29	11.2
Cu-02	45	60–100	31.7
Cu-03	0.5	80	14.0
Cu-04	12	>10	23.2
Ti-01 *^4^*	54	55.55	83.5
Ti-02	17		6.8
Ti-03	82		52.9
Ti-04	91		51.2
Ti-05	24		54.6
Ti-06	152	356	
Ti-07	46	40	41.8
Ti-08	28	40	39.7
Ti-09	41	10	44.1
Ti-10	16	10	37.8
Ti-11	24		
Zn-01	35	~65	48.6
Zn-02	29	>60	41.2
Zn-03	22	15	31.5
Zn-04	16	8	16.5
Zn-05	14	15	25.5
Zn-06	12		13.5
Zn-07	13		22.8

*^1^* Supplier data is not available for all metal oxides. ^*2*^ SSA calculated is obtained from the surface area calculated from the mean equivalent diameter measured by TEM and the material density, as provided by the manufacturer. For TiO_2_ samples, the density of the predominant phase (rutile or anatase) was used. *^3^* Two determinations. *^4^* Ti-01 is a NIST standard reference material with a certified value of 55.55 m^2^/g (± 0.70, expanded uncertainty) for specific surface area.

## Supplementary Materials

The following are available online at https://www.mdpi.com/2079-4991/10/9/1812/s1, Figures S1–S29 with XRD data and Figures S30–S38 with TEM images and particle size histograms for all samples.
