# Supplementary material for: Characterization of Commercial Metal Oxide Nanomaterials: Crystalline Phase, Particle Size and Specific Surface Area
Source: Nanomaterials (Basel). 2020 Sep 11;10(9):1812. doi: 10.3390/nano10091812 (PMC7558088; doi:10.3390/nano10091812)
Supplement: Supplementary file 1 [file nanomaterials-10-01812-s001.pdf]

# Supplementary Materials

## Characterization of Commercial Metal Oxide Nanomaterials: Crystalline Phase, Particle Size and Specific Surface Area

**Michael Bushell, Suzanne Beauchemin *, Filip Kunc, David Gardner, Jeffrey Ovens, Floyd Toll, David Kennedy, Kathy Nguyen, Djordje Vladisavljevic, Pat Rasmussen, Linda J. Johnston ,***

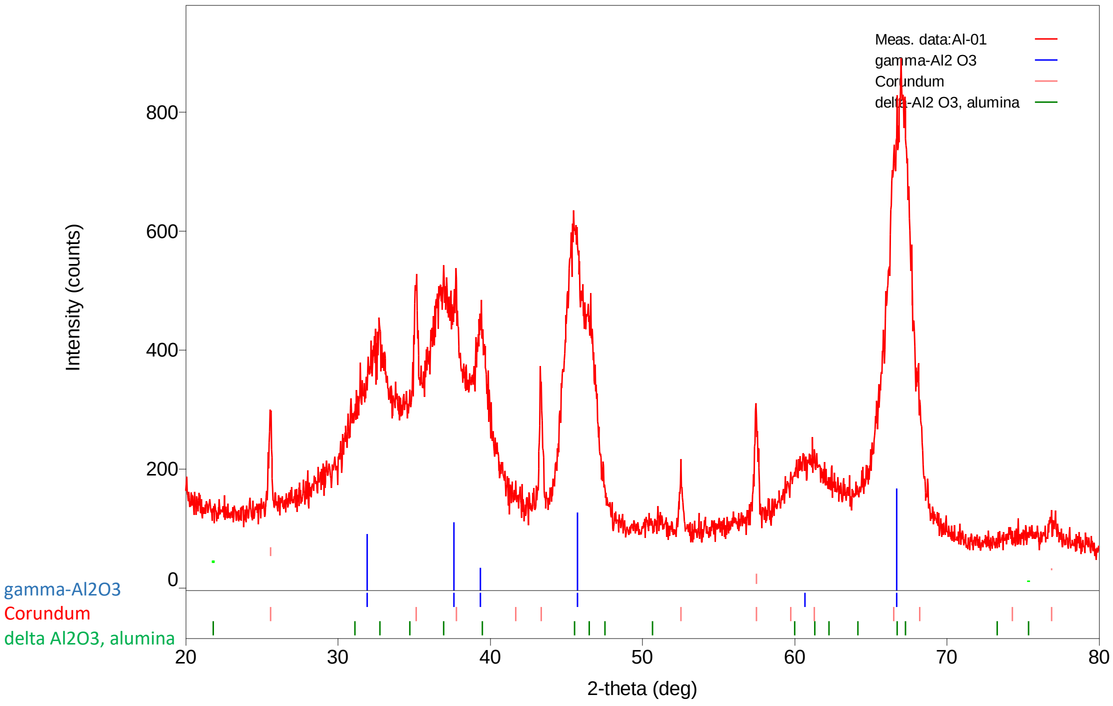


**Figure S1**. X-ray diffraction pattern for the sample Al-01, along with relevant reference compounds.

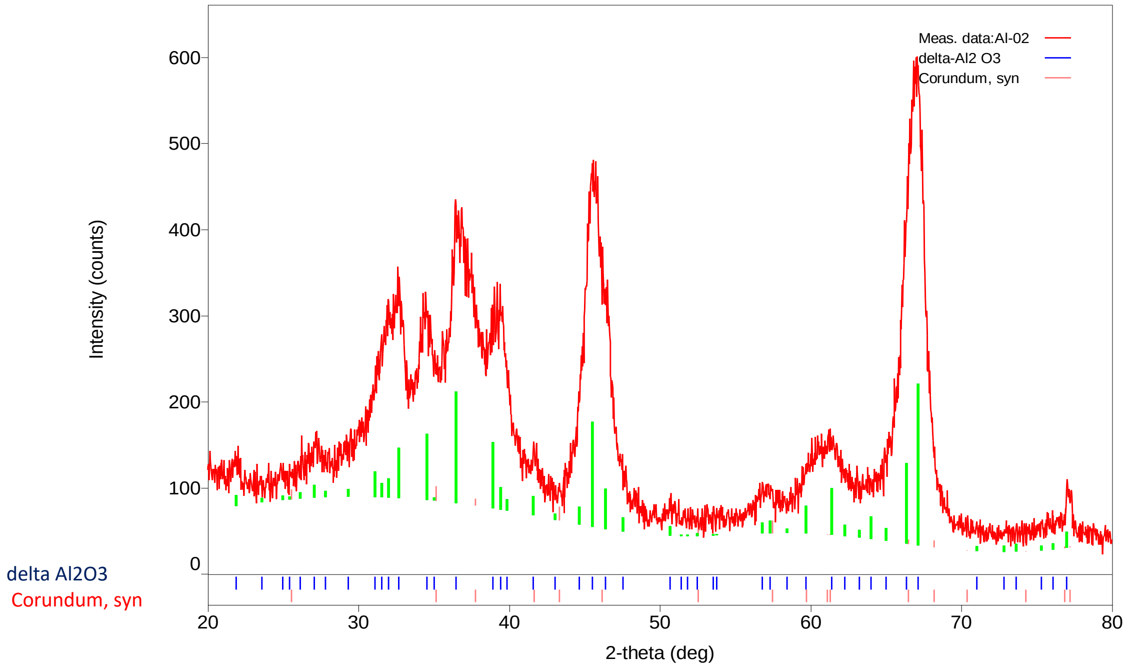


**Figure S2**. X-ray diffraction pattern for the sample Al-02, along with relevant reference compounds.

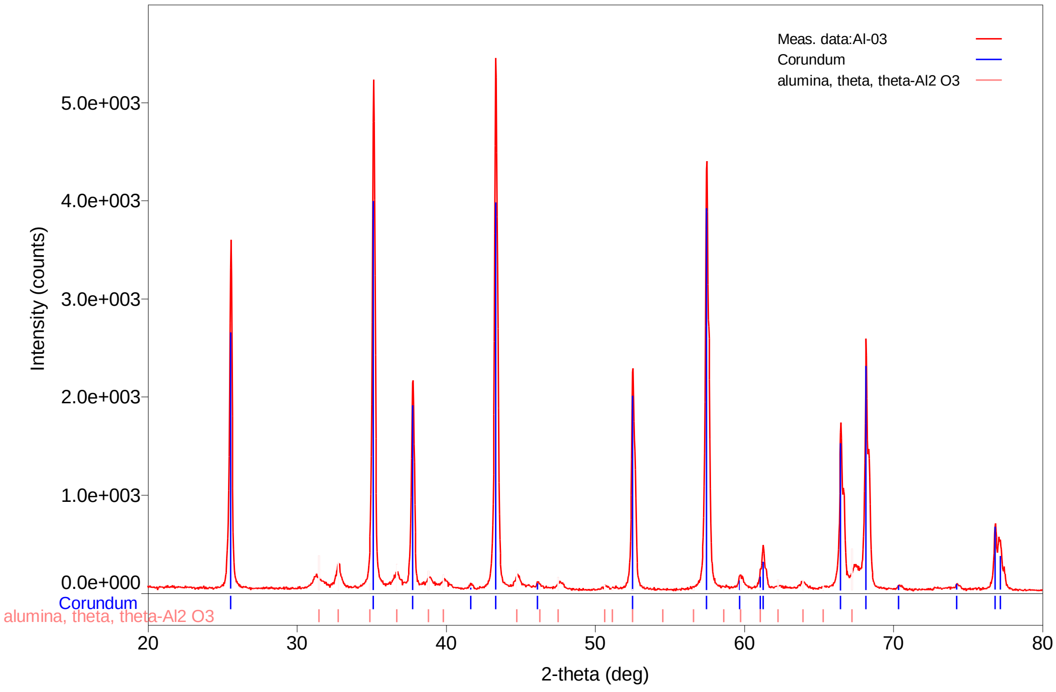


**Figure S3**. X-ray diffraction pattern for the sample Al-03, along with relevant reference compounds.

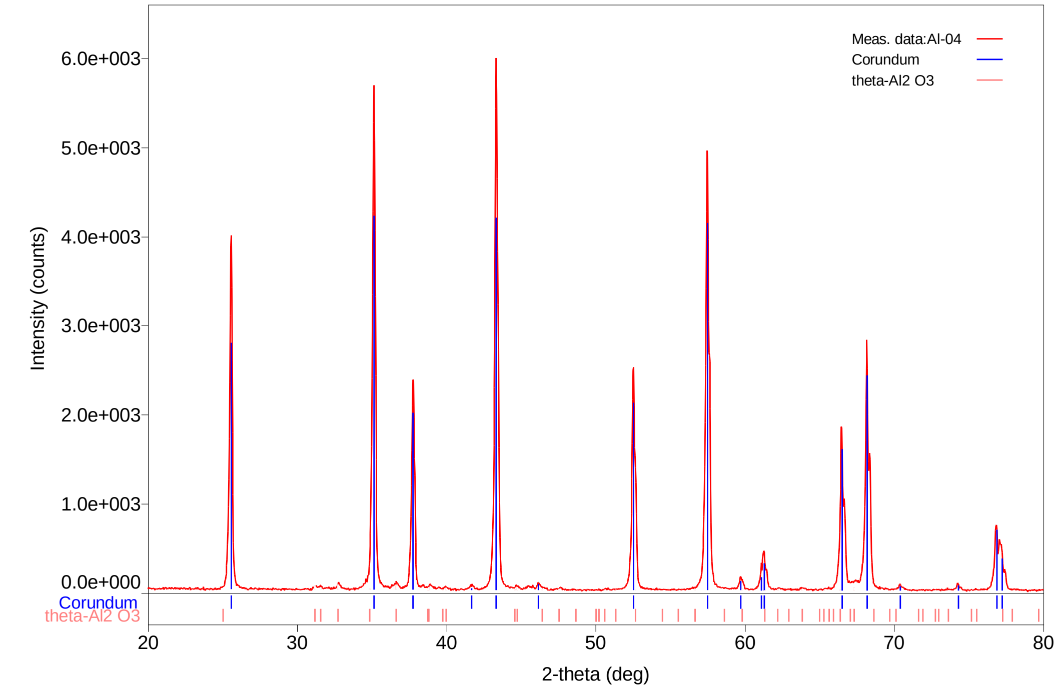


**Figure S4**. X-ray diffraction pattern for the sample Al-04, along with relevant reference compounds.

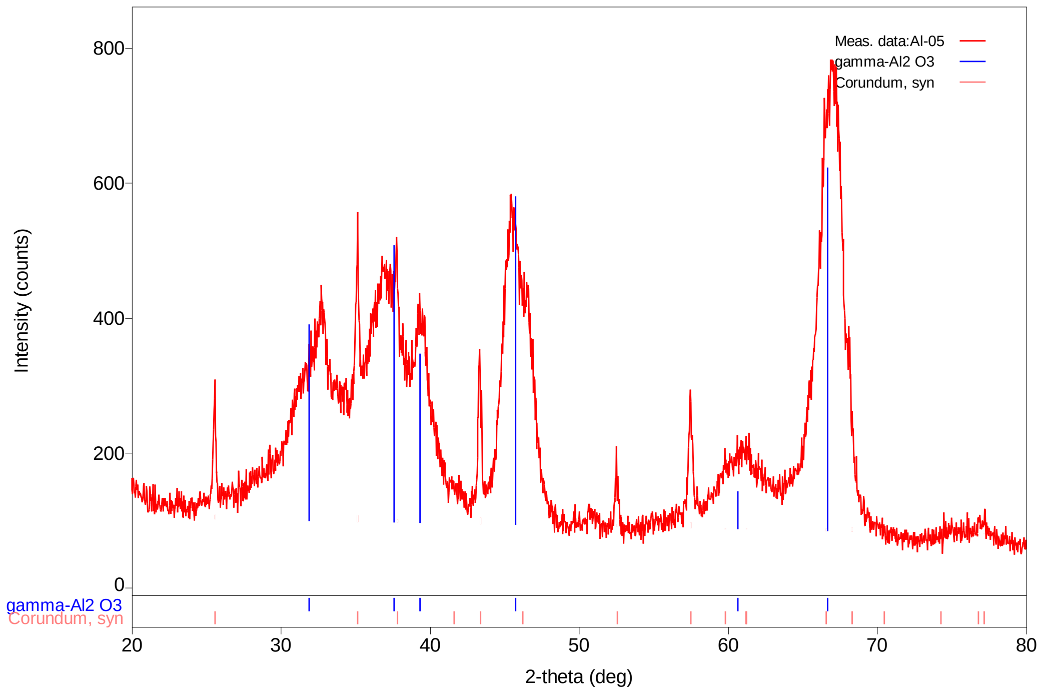


**Figure S5**. X-ray diffraction pattern for the sample Al-05, along with relevant reference compounds.

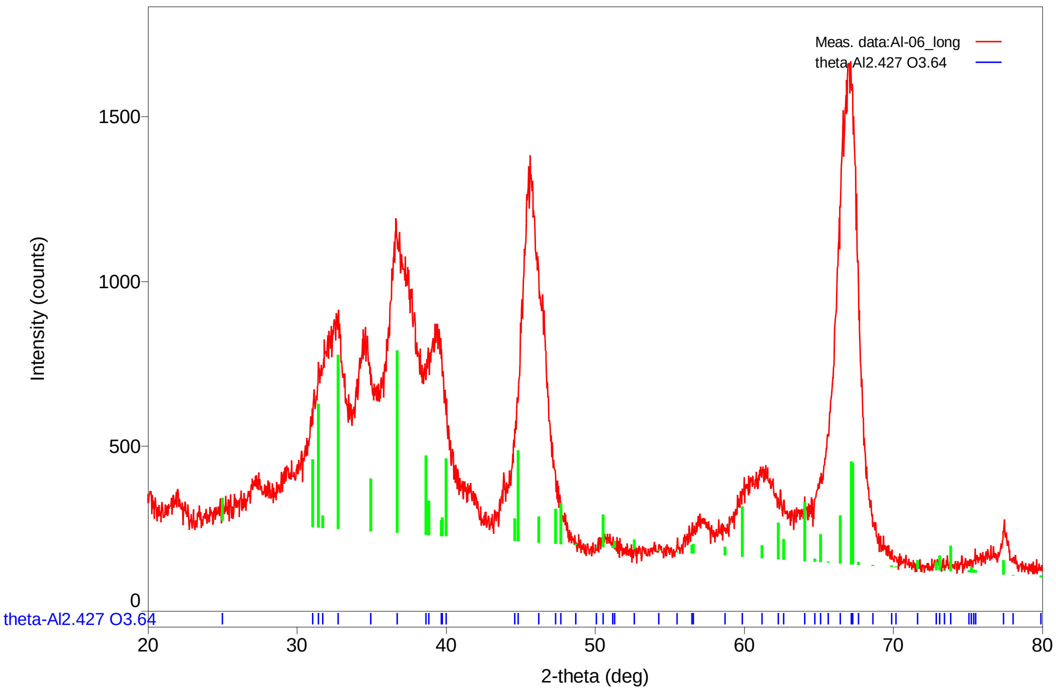


**Figure S6.** X-ray diffraction pattern for the sample Al-06, along with relevant reference compounds (longer scan using 0.3 deg/min for refined analysis).

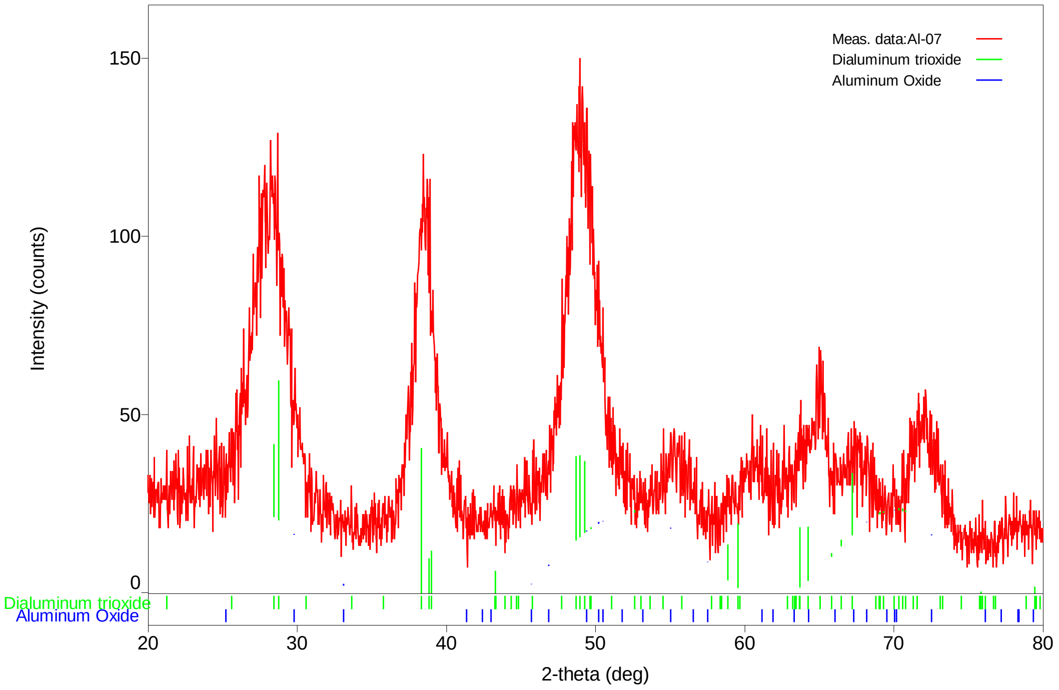


**Figure S7**. X-ray diffraction pattern for the sample Al-07, along with relevant reference compounds.

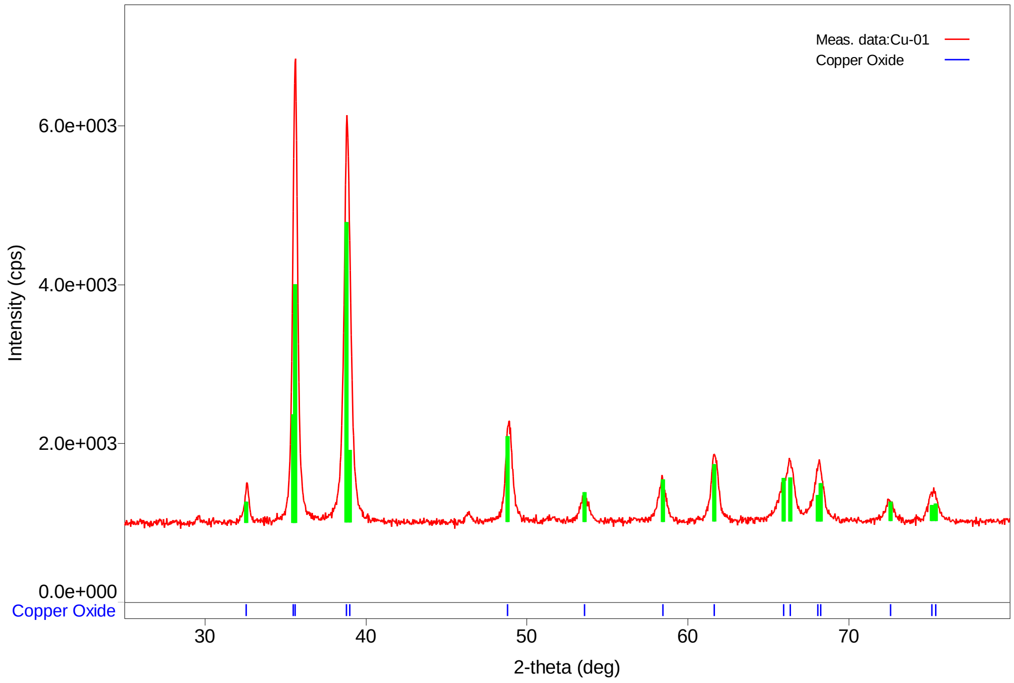


**Figure S8**. X-ray diffraction pattern for the sample Cu-01, along with relevant reference compounds.

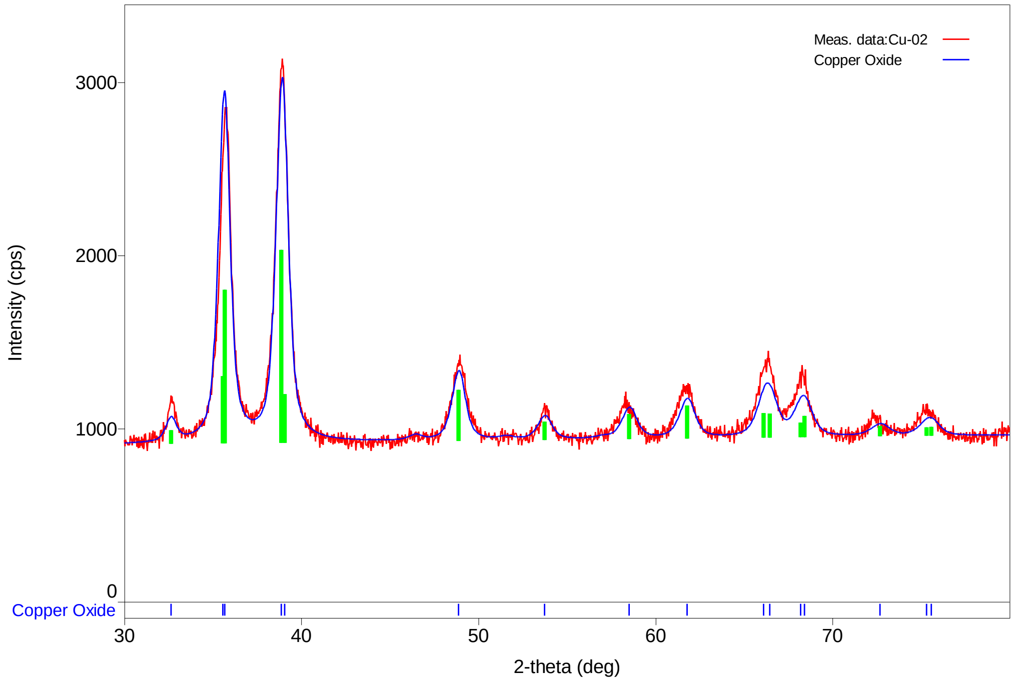


**Figure S9**. X-ray diffraction pattern for the sample Cu-02, along with relevant reference compounds.

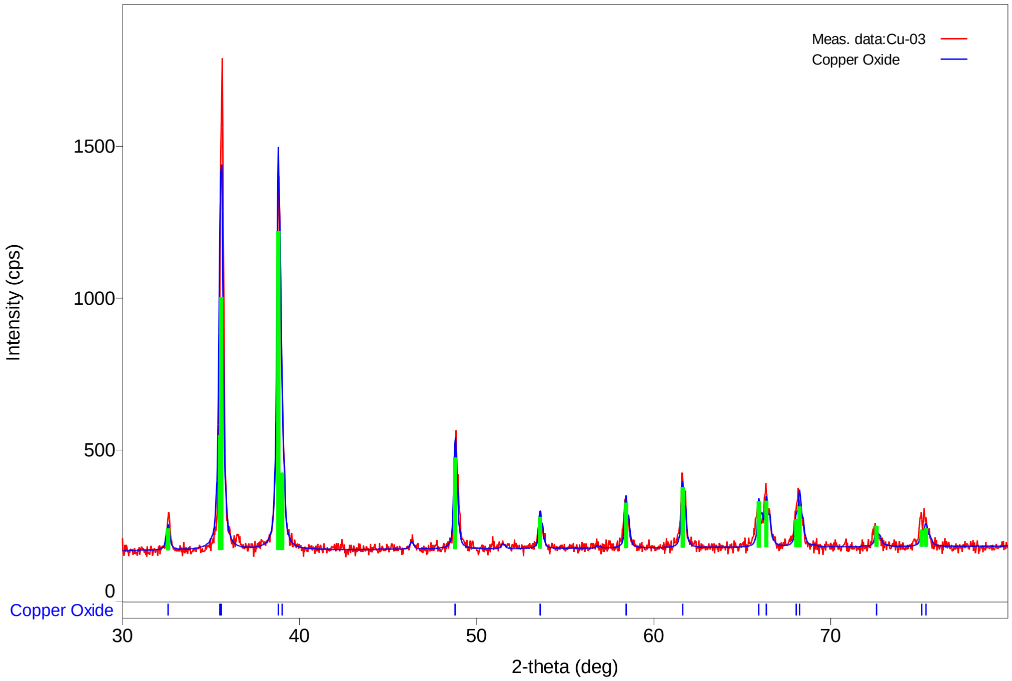


**Figure S10**. X-ray diffraction pattern for the sample Cu-03, along with relevant reference compounds.

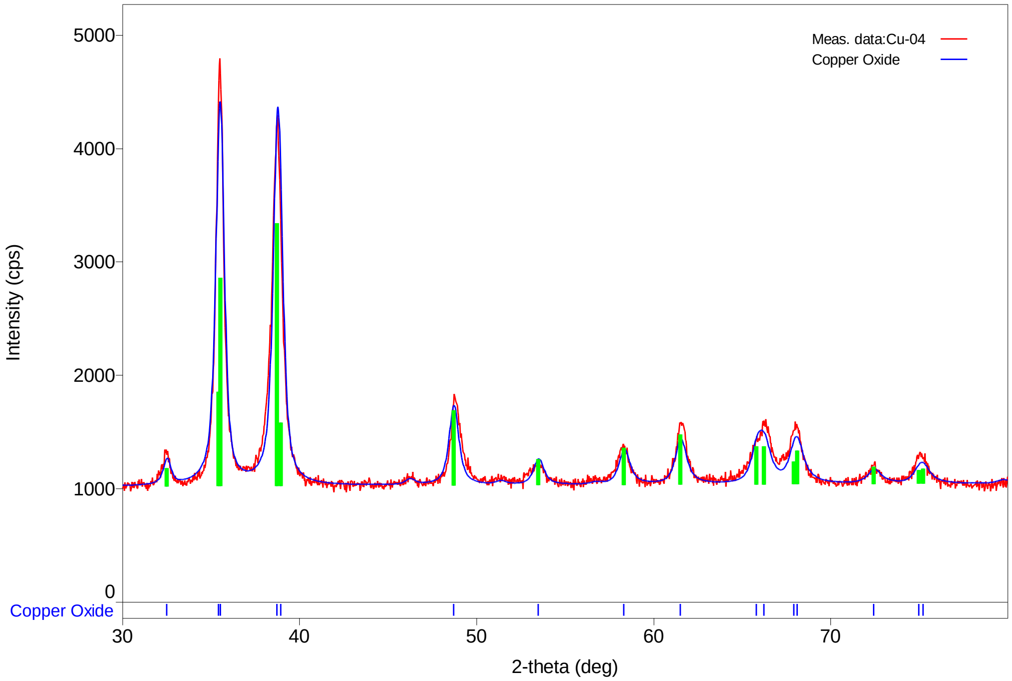


**Figure S11**. X-ray diffraction pattern for the sample Cu-04, along with relevant reference compounds.

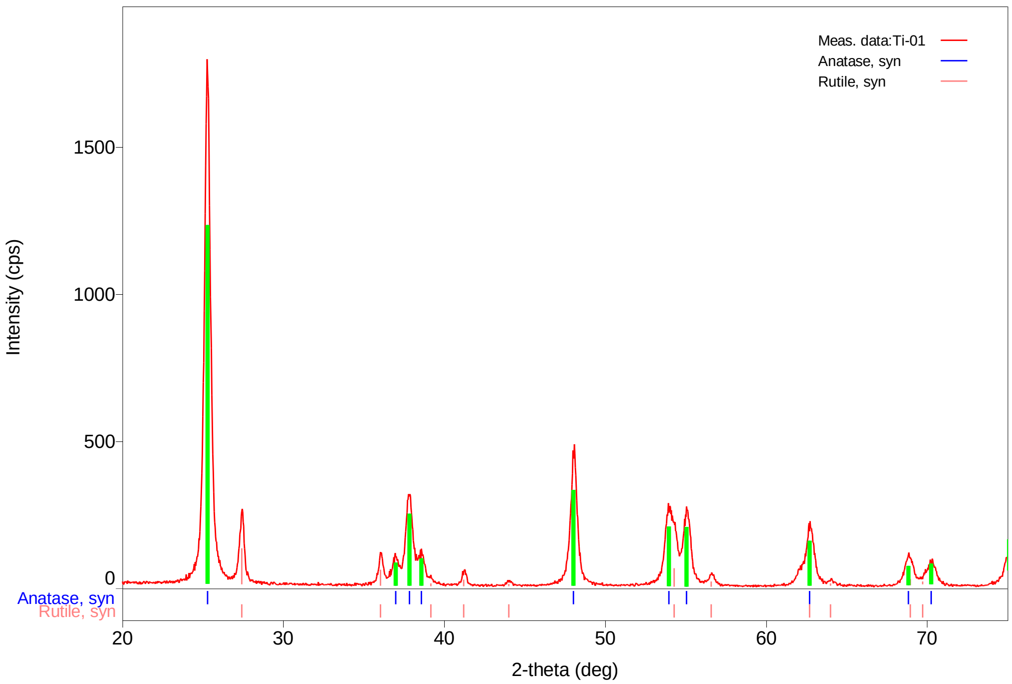


**Figure S12**. X-ray diffraction pattern for the sample Ti-01, along with relevant reference compounds.

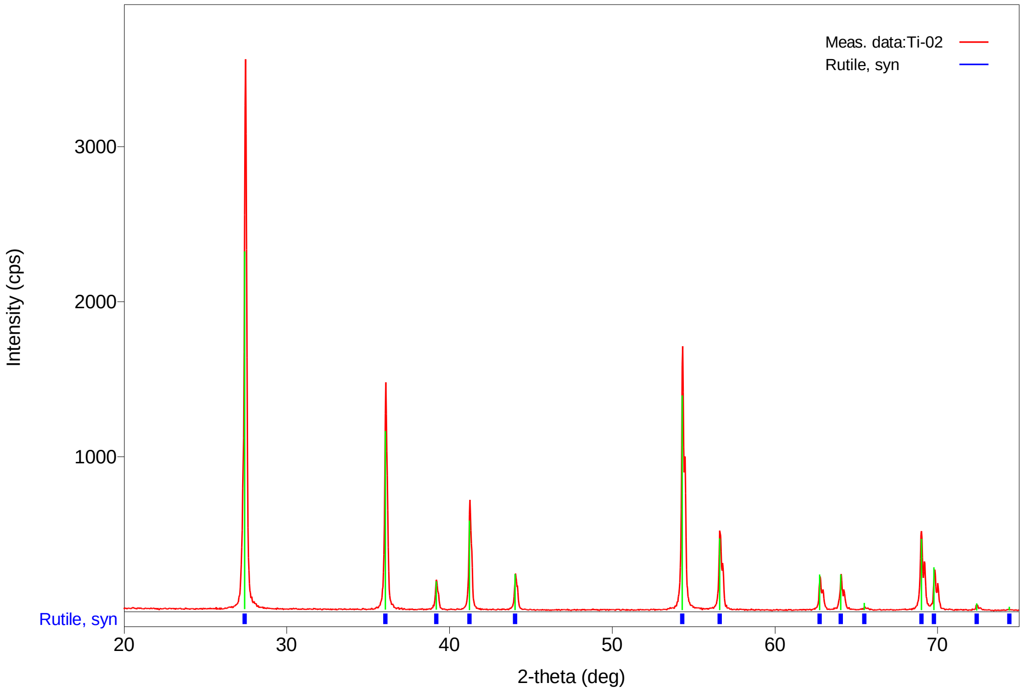


**Figure S13**. X-ray diffraction pattern for the sample Ti-02, along with relevant reference compounds.

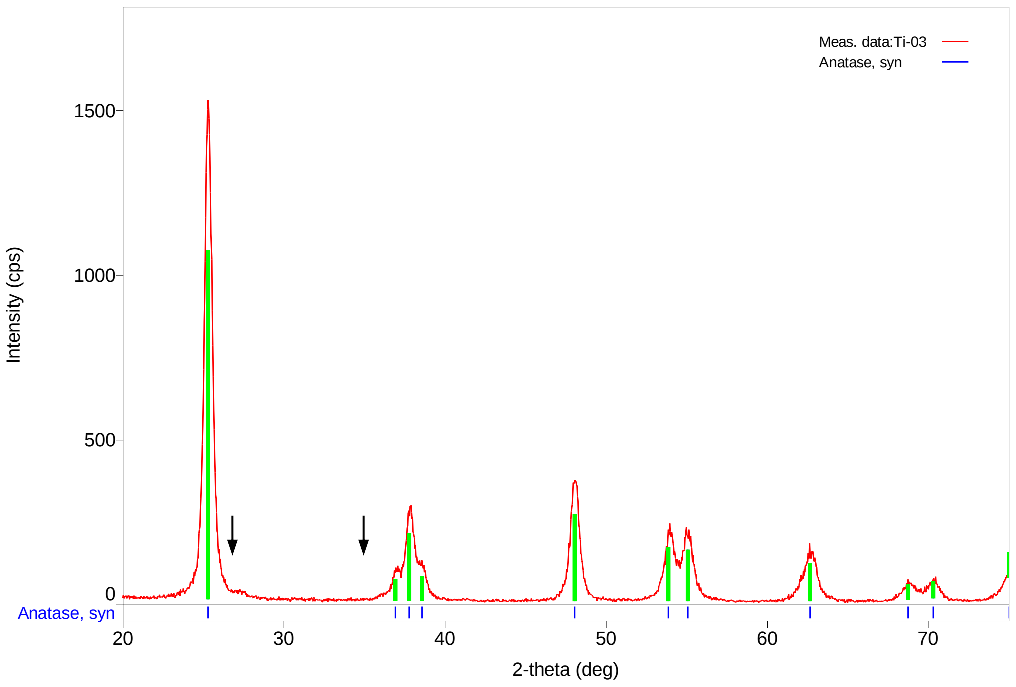


**Figure S14**. X-ray diffraction pattern for the sample Ti-03, along with relevant reference compounds; black arrows indicate the occurrence of trace amounts of rutile.

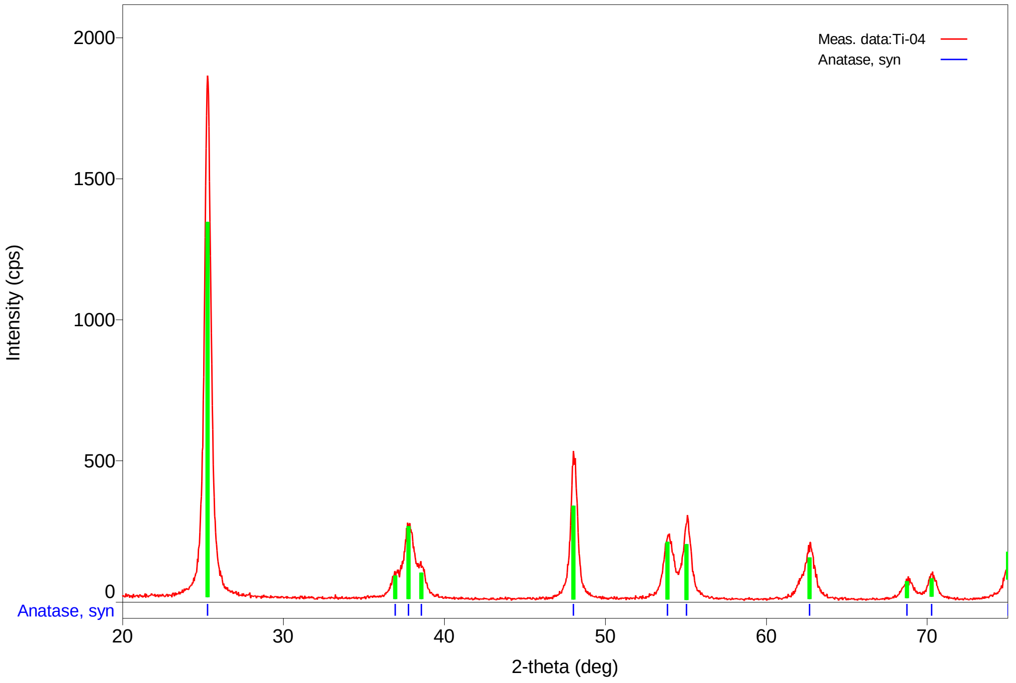


**Figure S15**. X-ray diffraction pattern for the sample Ti-04, along with relevant reference compounds.

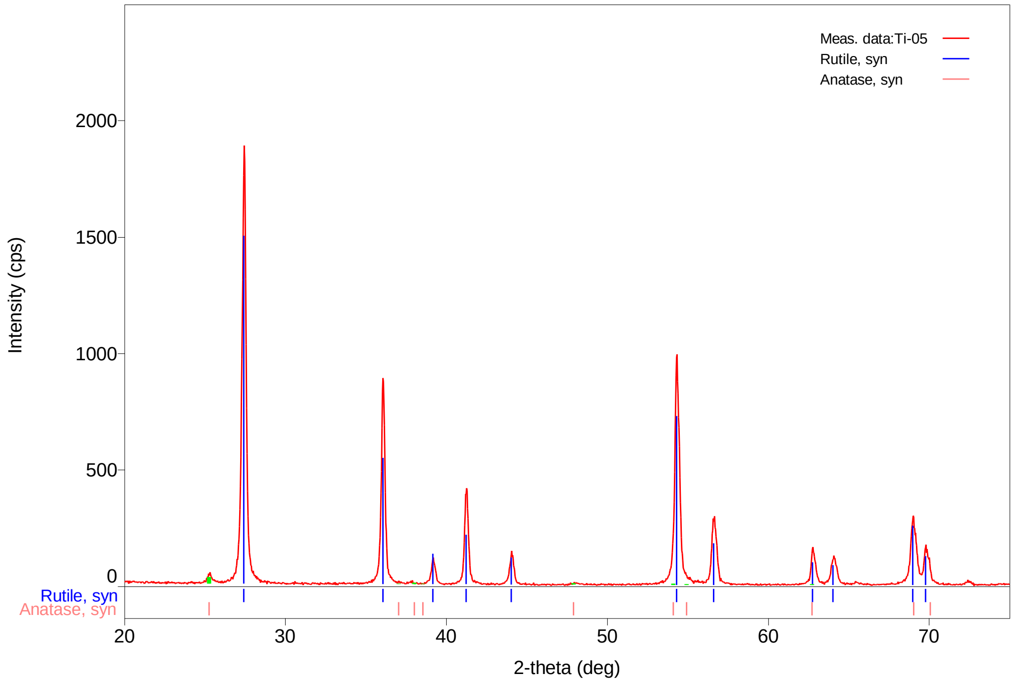


**Figure S16**. X-ray diffraction pattern for the sample Ti-05, along with relevant reference compounds.

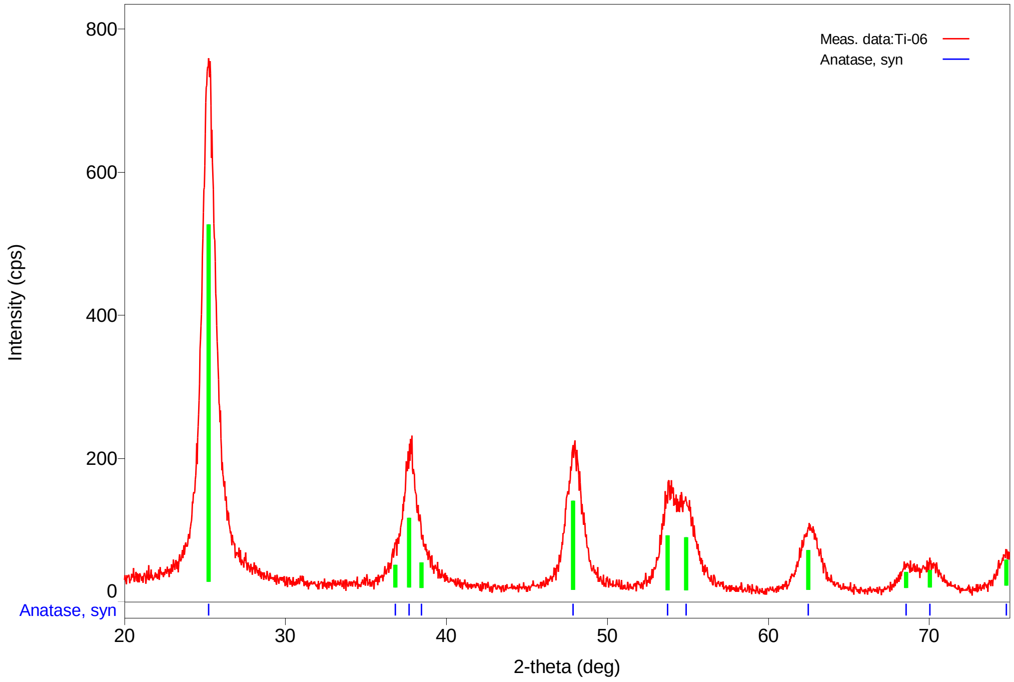


**Figure S17**. X-ray diffraction pattern for the sample Ti-06, along with relevant reference compounds.

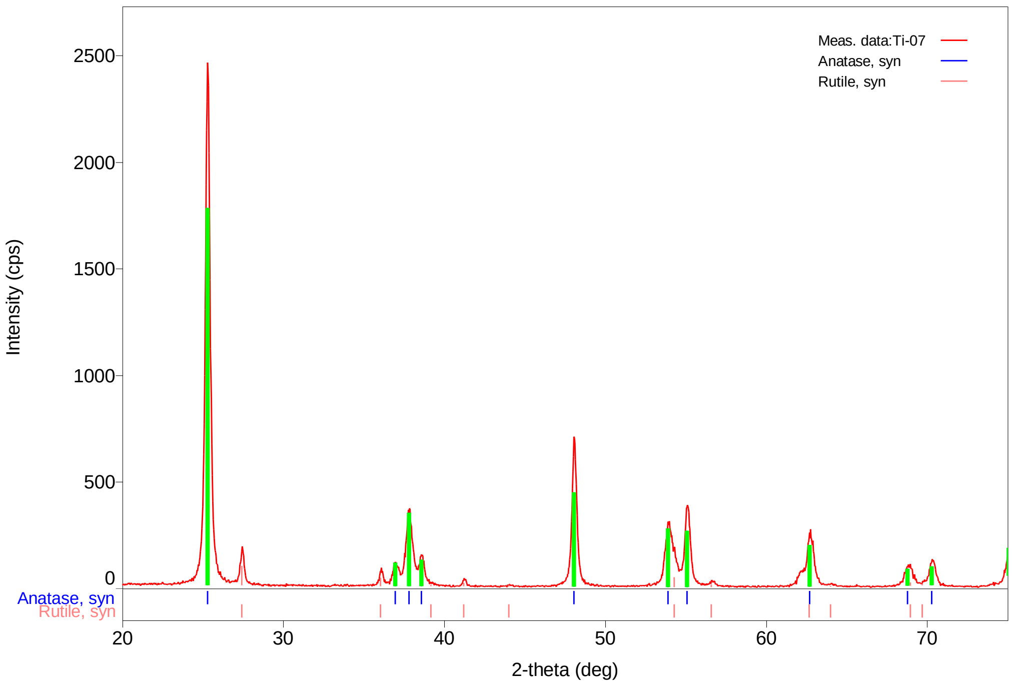


**Figure S18**. X-ray diffraction pattern for the sample Ti-07, along with relevant reference compounds.

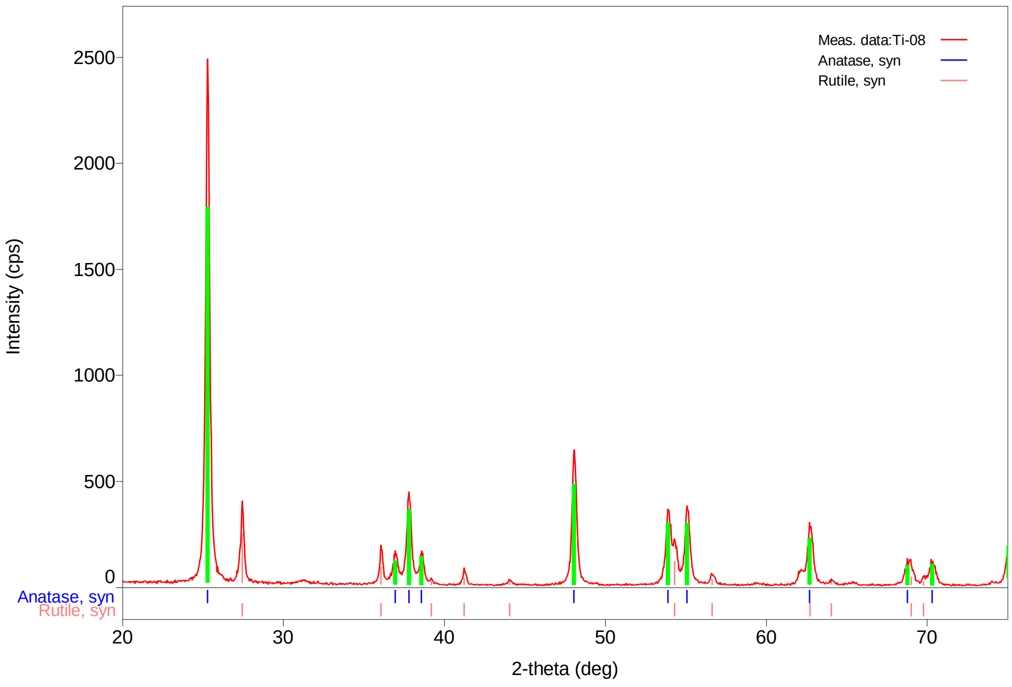


**Figure S19**. X-ray diffraction pattern for the sample Ti-08, along with relevant reference compounds.

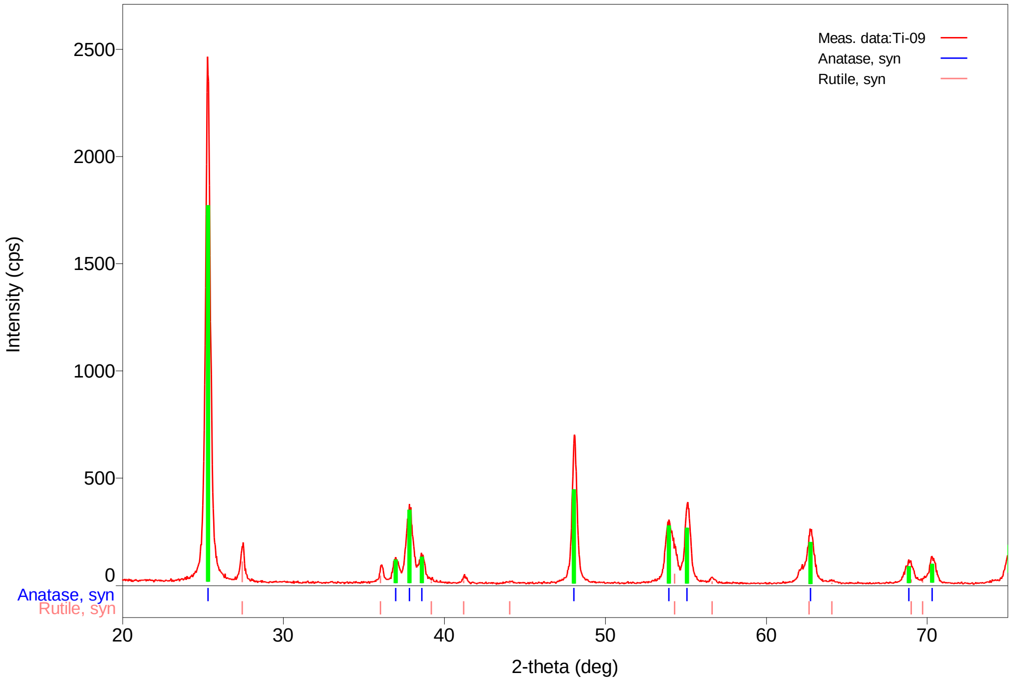


**Figure S20**. X-ray diffraction pattern for the sample Ti-09, along with relevant reference compounds.

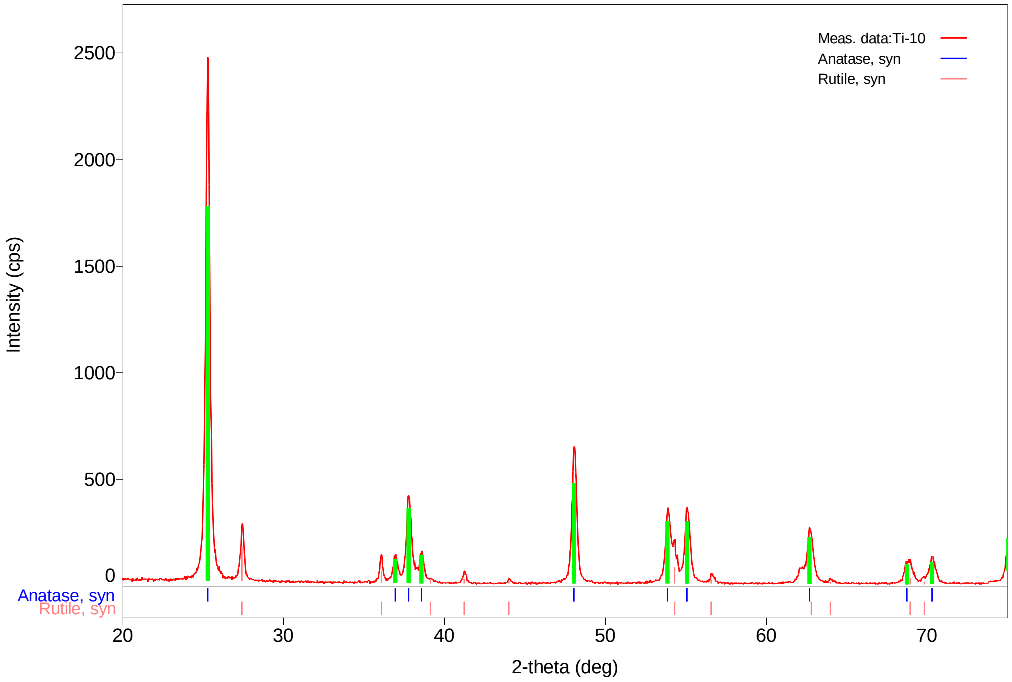


**Figure S21**. X-ray diffraction pattern for the sample Ti-10, along with relevant reference compounds.

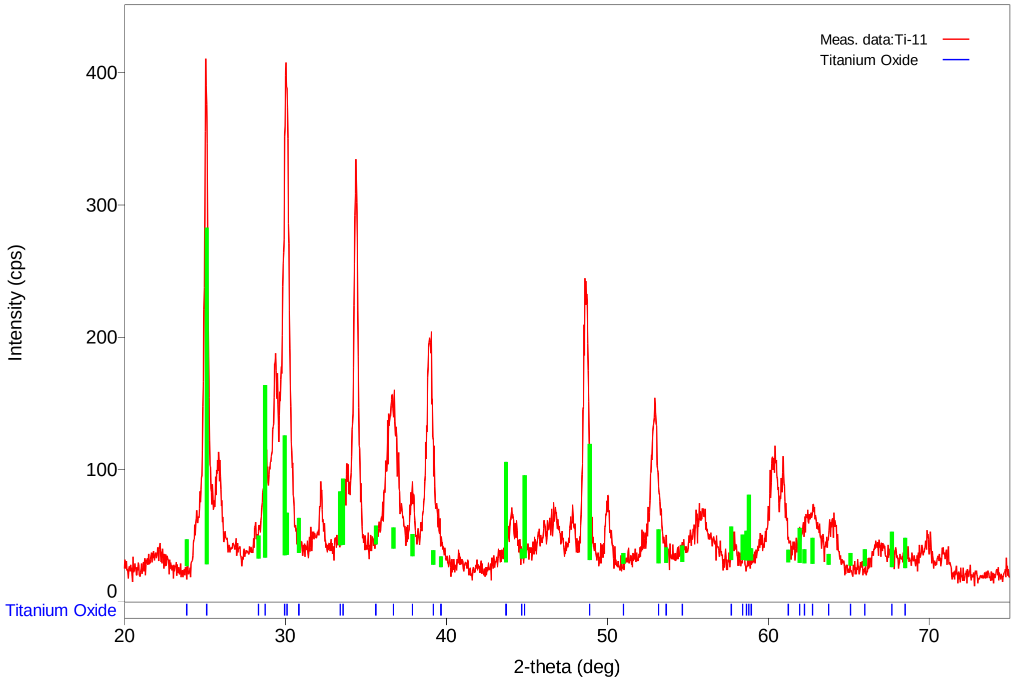


**Figure S22**. X-ray diffraction pattern for the sample Ti-11, along with relevant reference compounds.

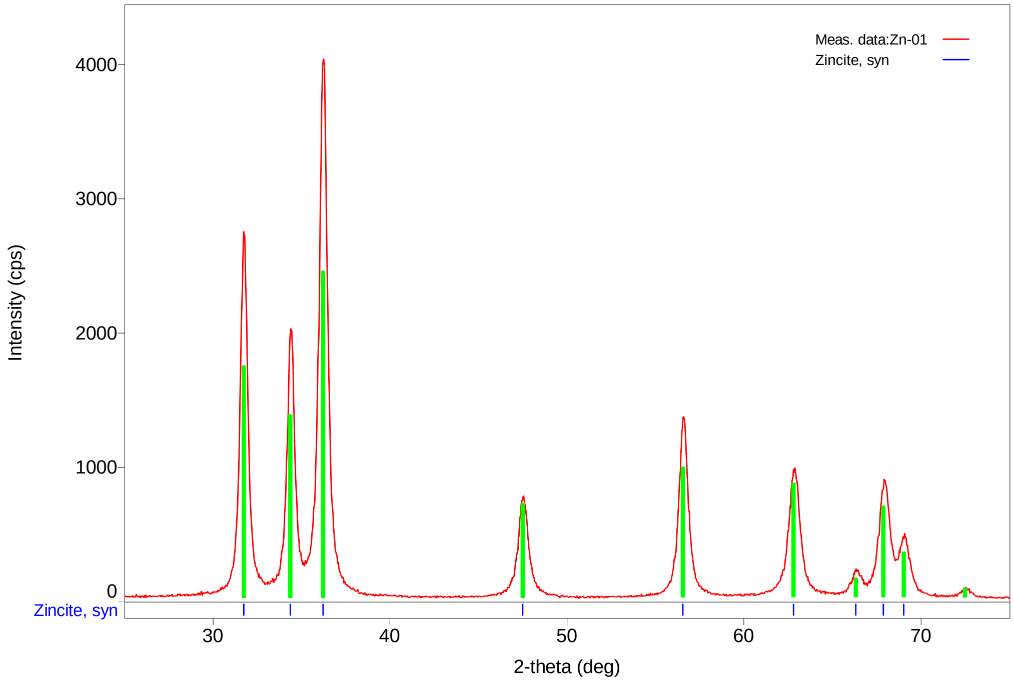


**Figure S23**. X-ray diffraction pattern for the sample Zn-01, along with relevant reference compounds.

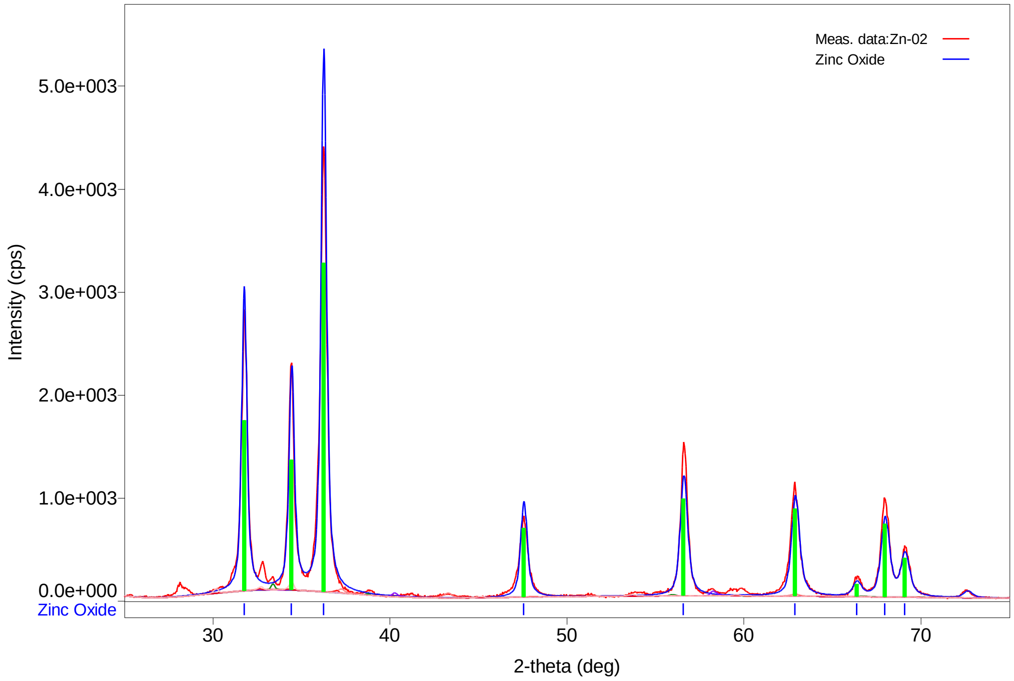


**Figure S24**. X-ray diffraction pattern for the sample Zn-02, along with relevant reference compounds.

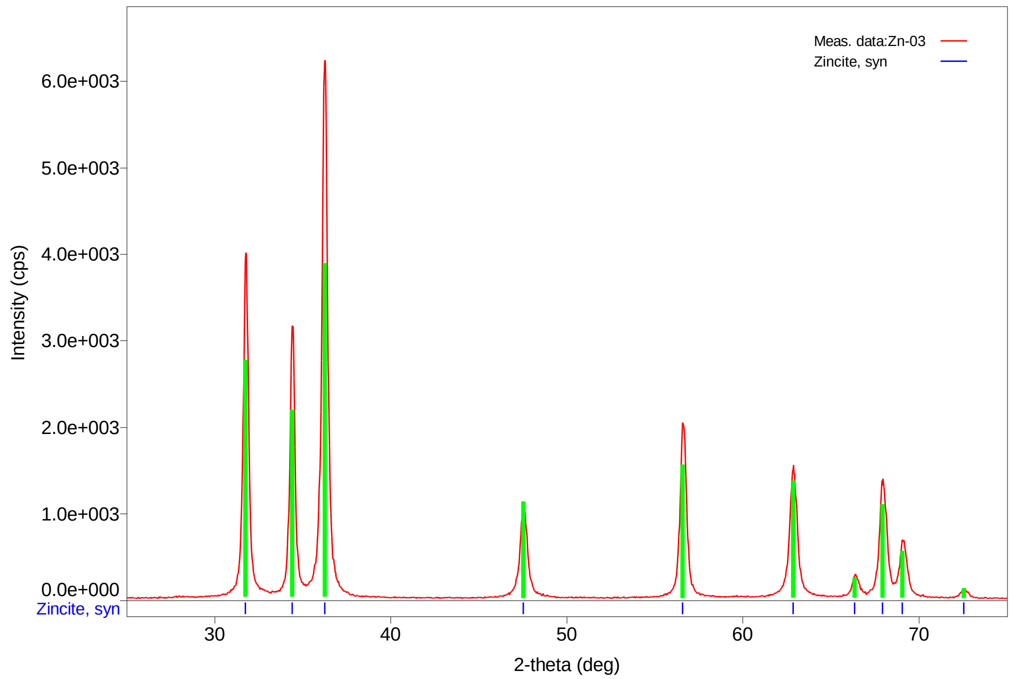


**Figure S25**. X-ray diffraction pattern for the sample Zn-03, along with relevant reference compounds.

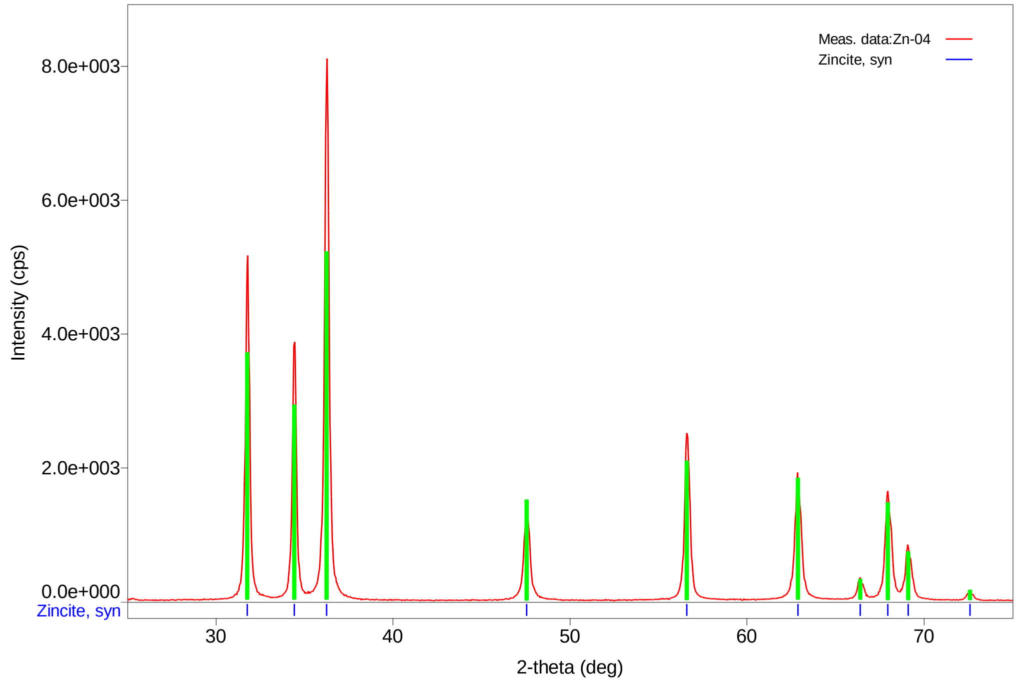


**Figure S26**. X-ray diffraction pattern for the sample Zn-04, along with relevant reference compounds.

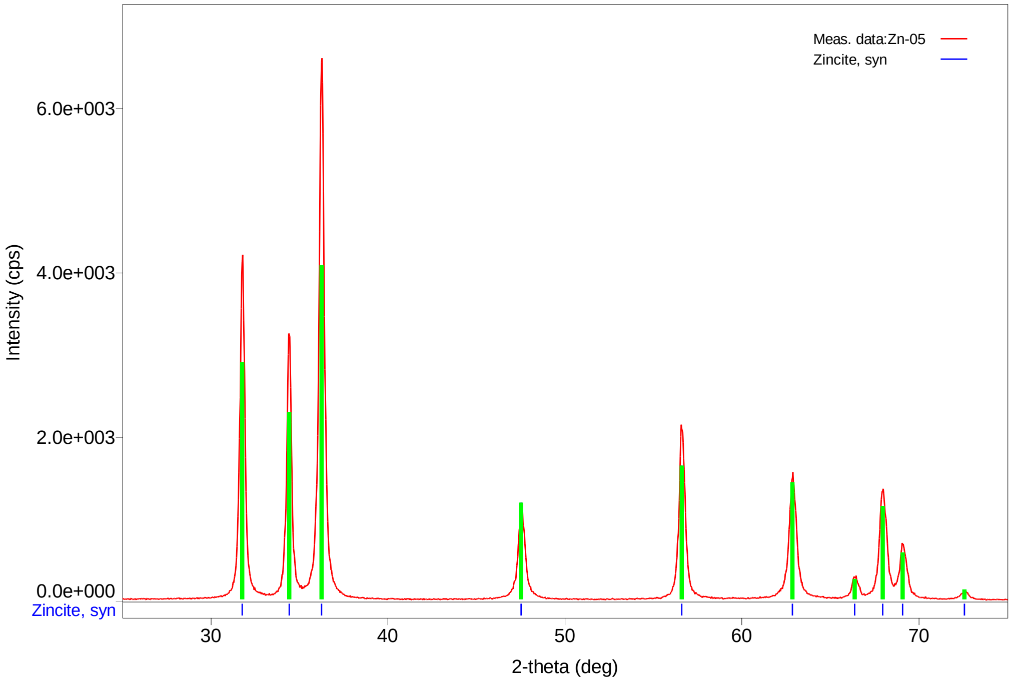


**Figure S27**. X-ray diffraction pattern for the sample Zn-05, along with relevant reference compounds.

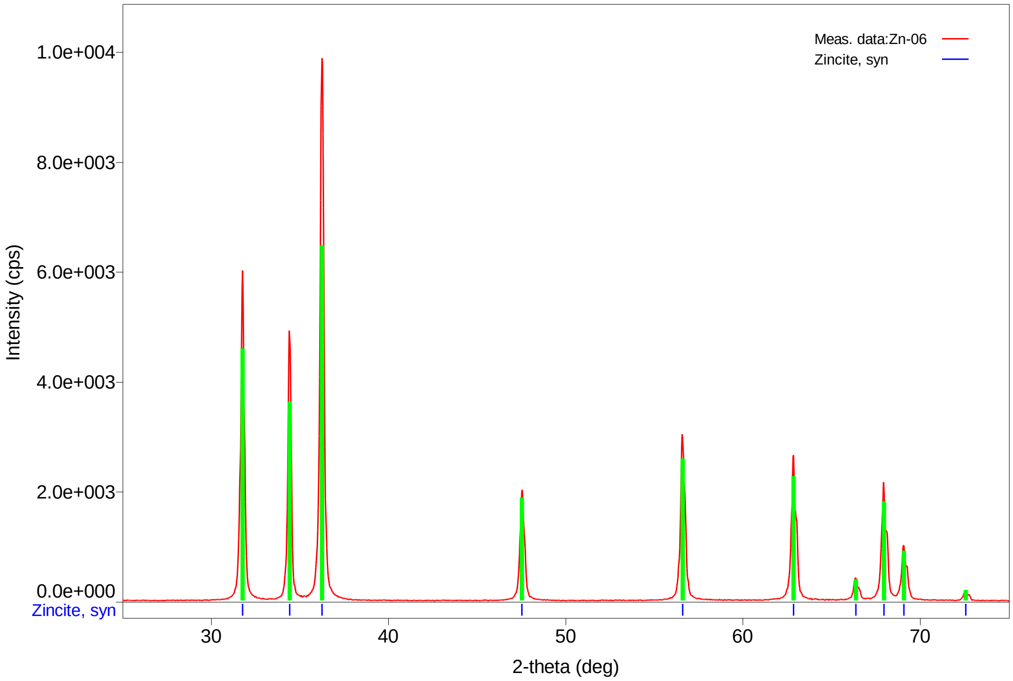


**Figure S28**. X-ray diffraction pattern for the sample Zn-06, along with relevant reference compounds.

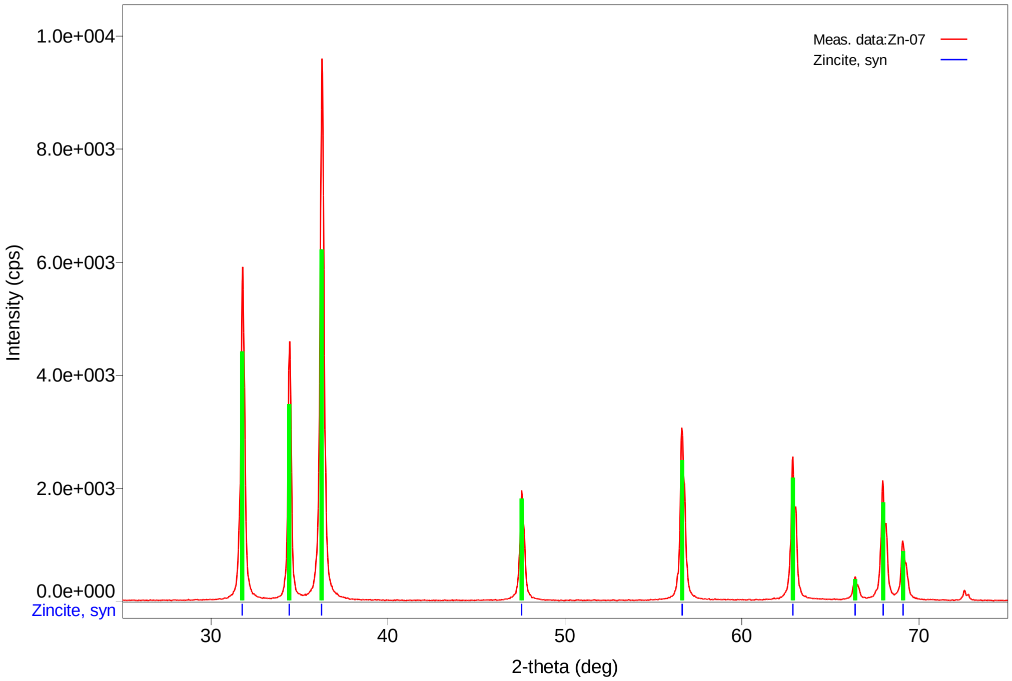


**Figure S29**. X-ray diffraction pattern for the sample Zn-07, along with relevant reference compounds.

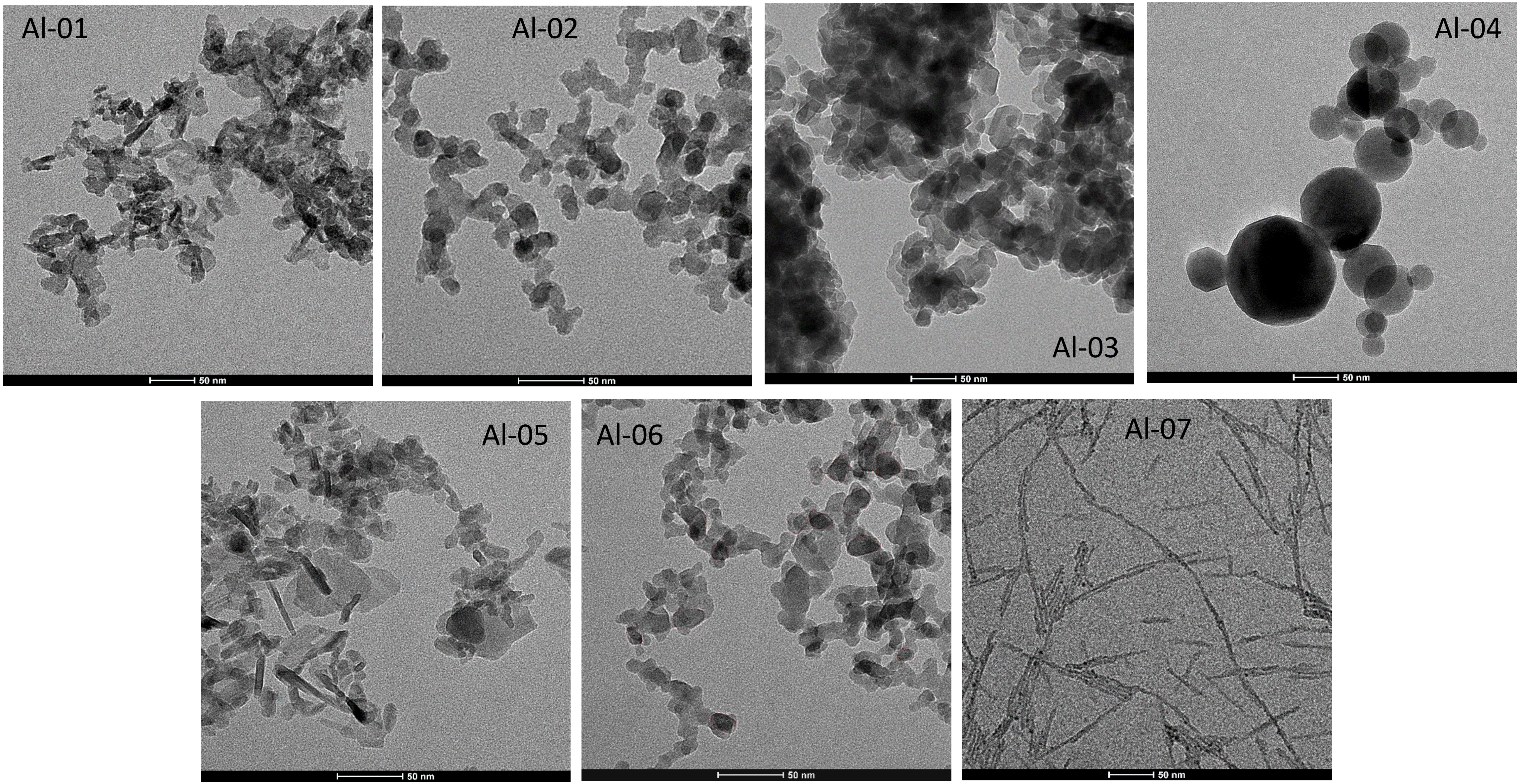


**Figure S30**.  TEM images of aluminum oxide nanomaterials

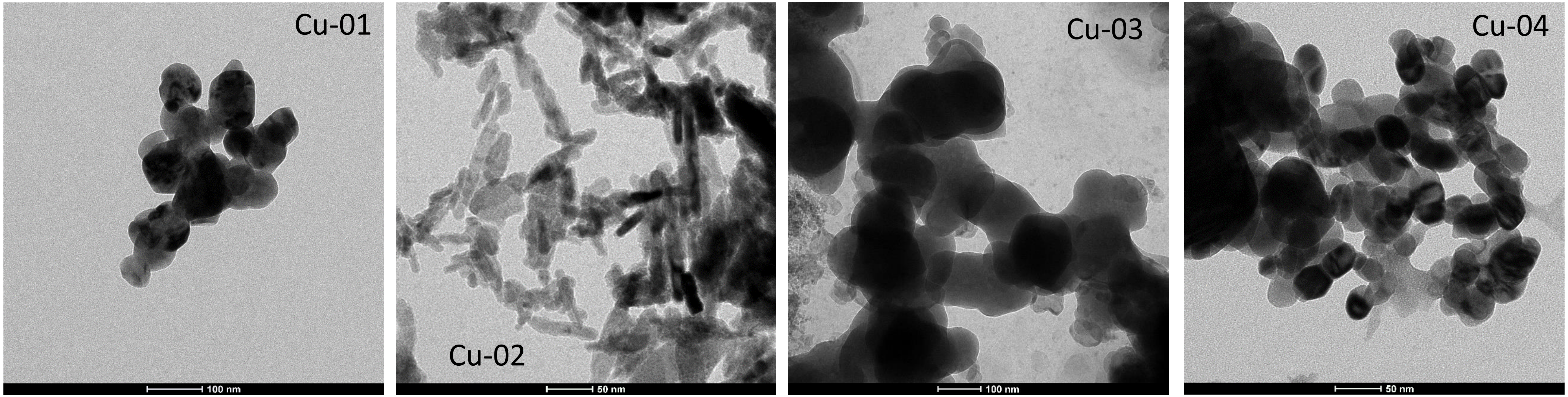


**Figure S31**. TEM images of copper oxide nanomaterials

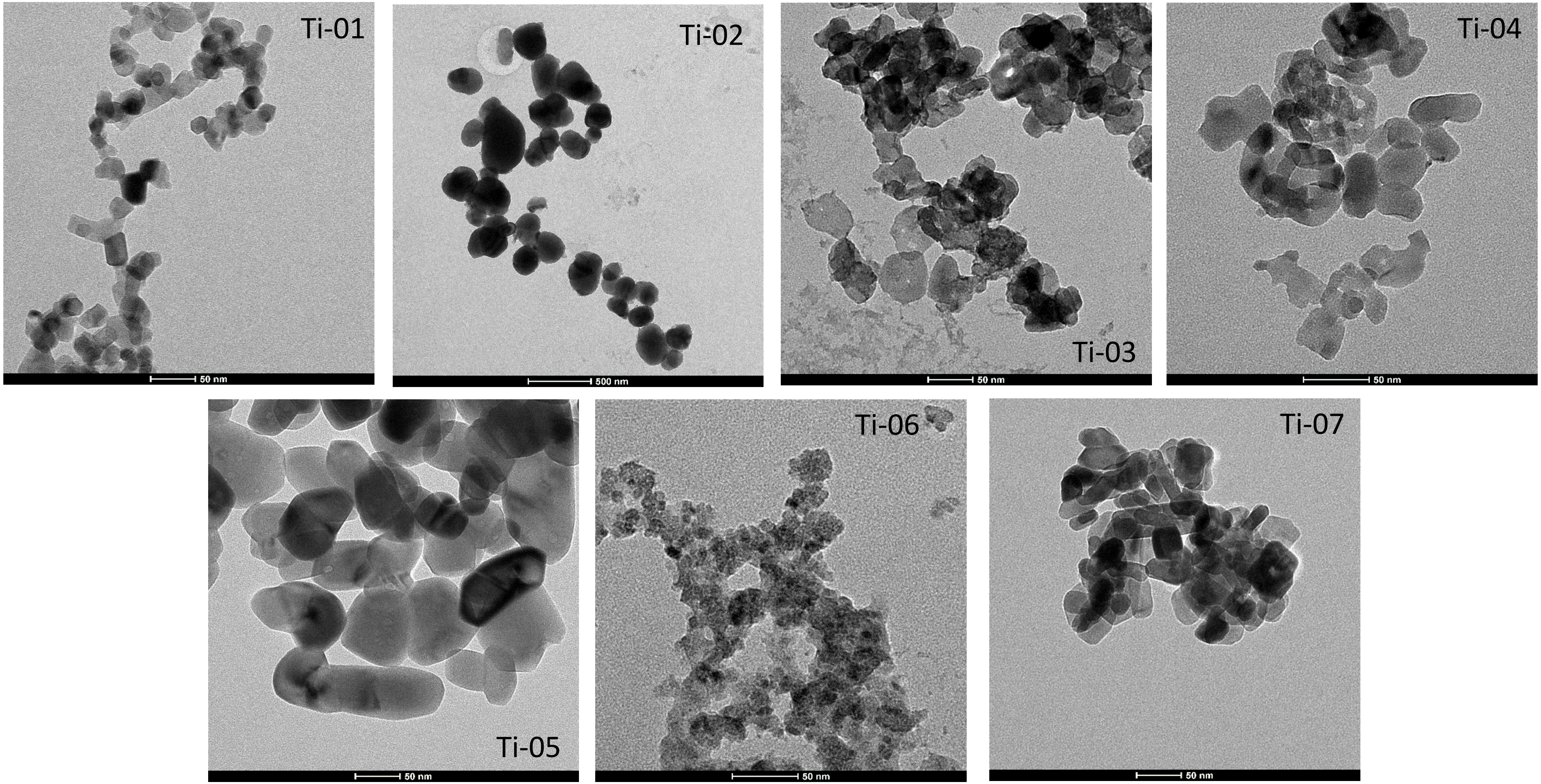


**Figure S32**. TEM images of titanium oxide nanomaterials

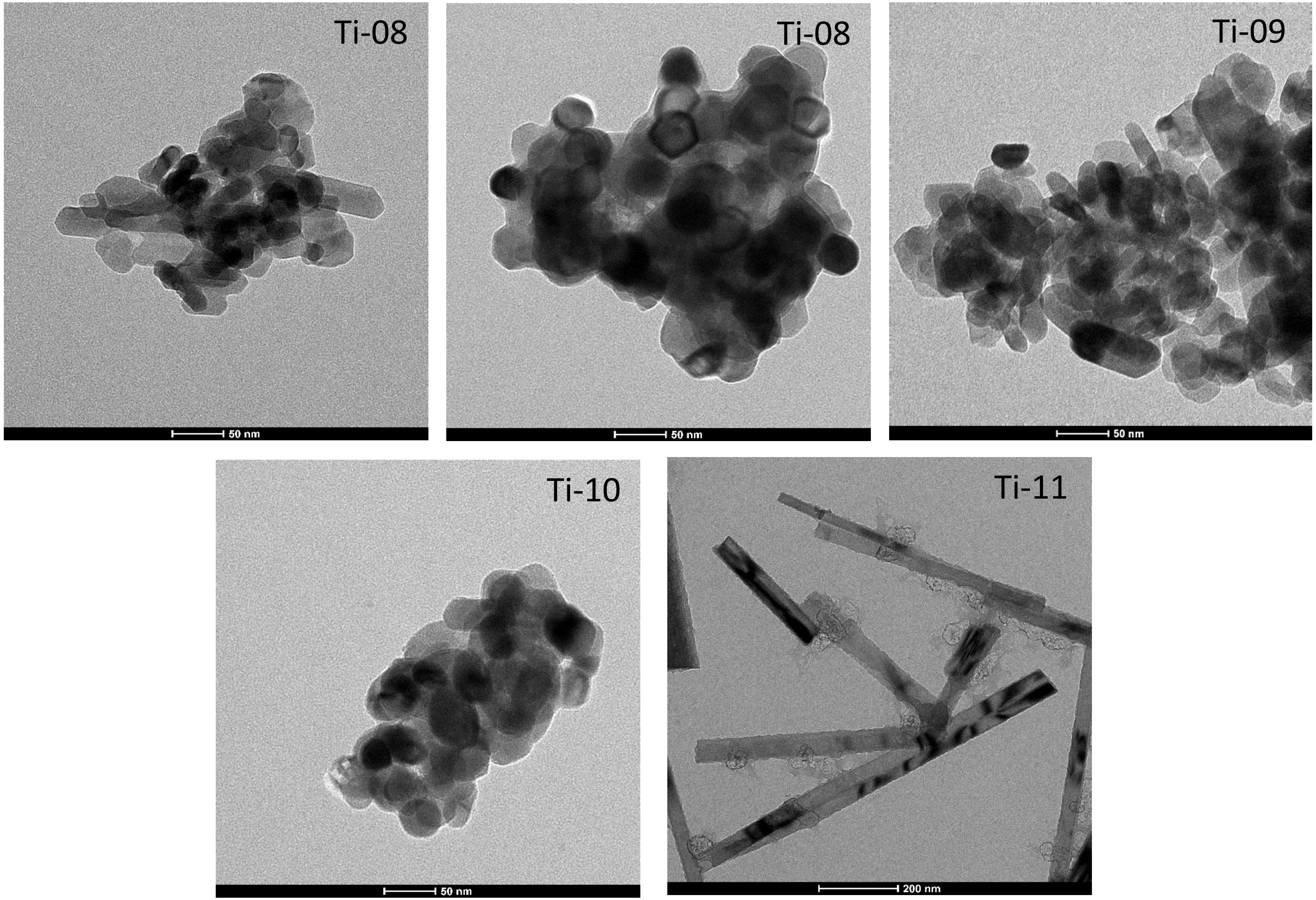


**Figure S33**. TEM images of titanium oxide nanomaterials

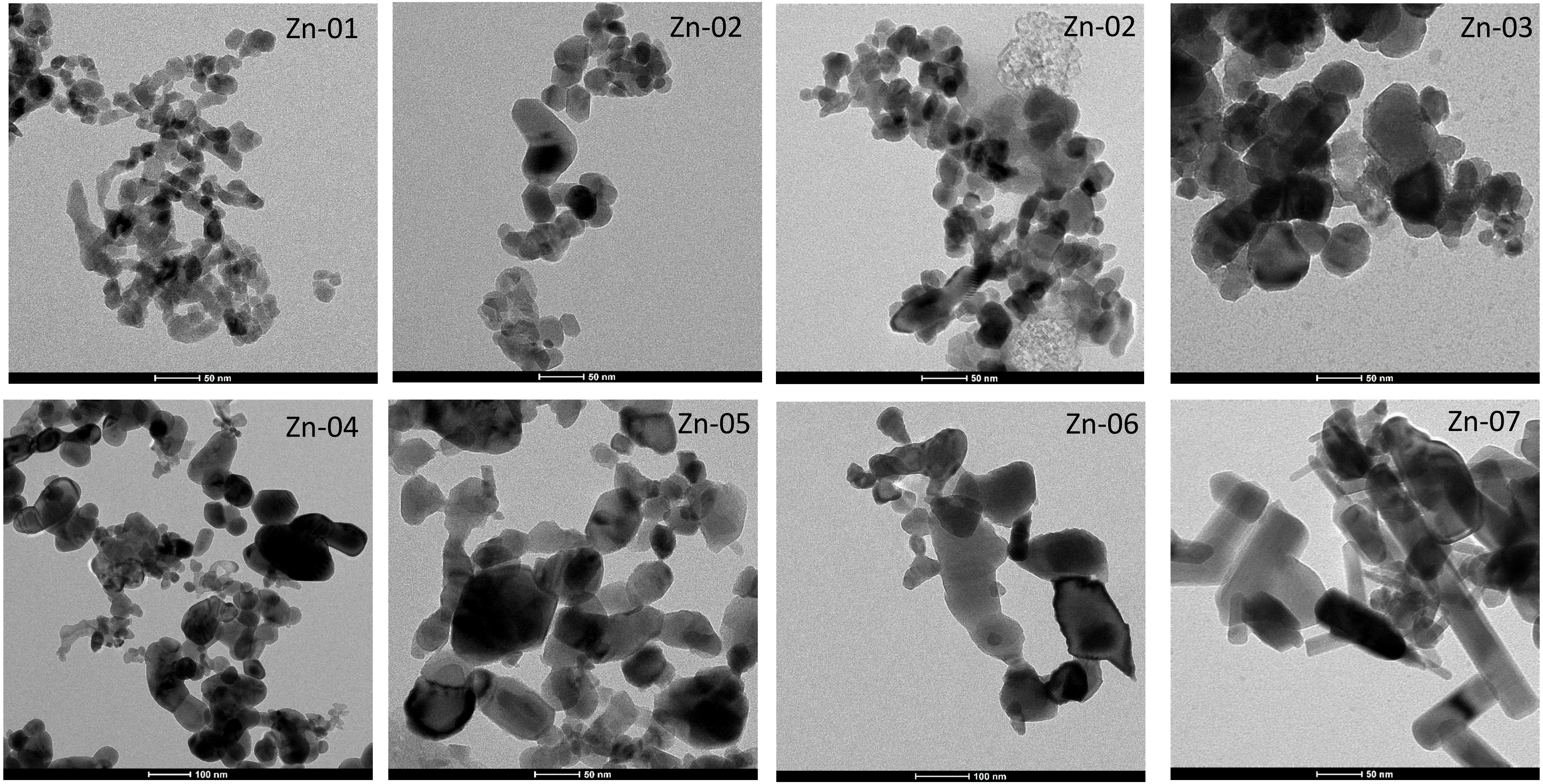


**Figure S34**. TEM images of zinc oxide nanomaterials

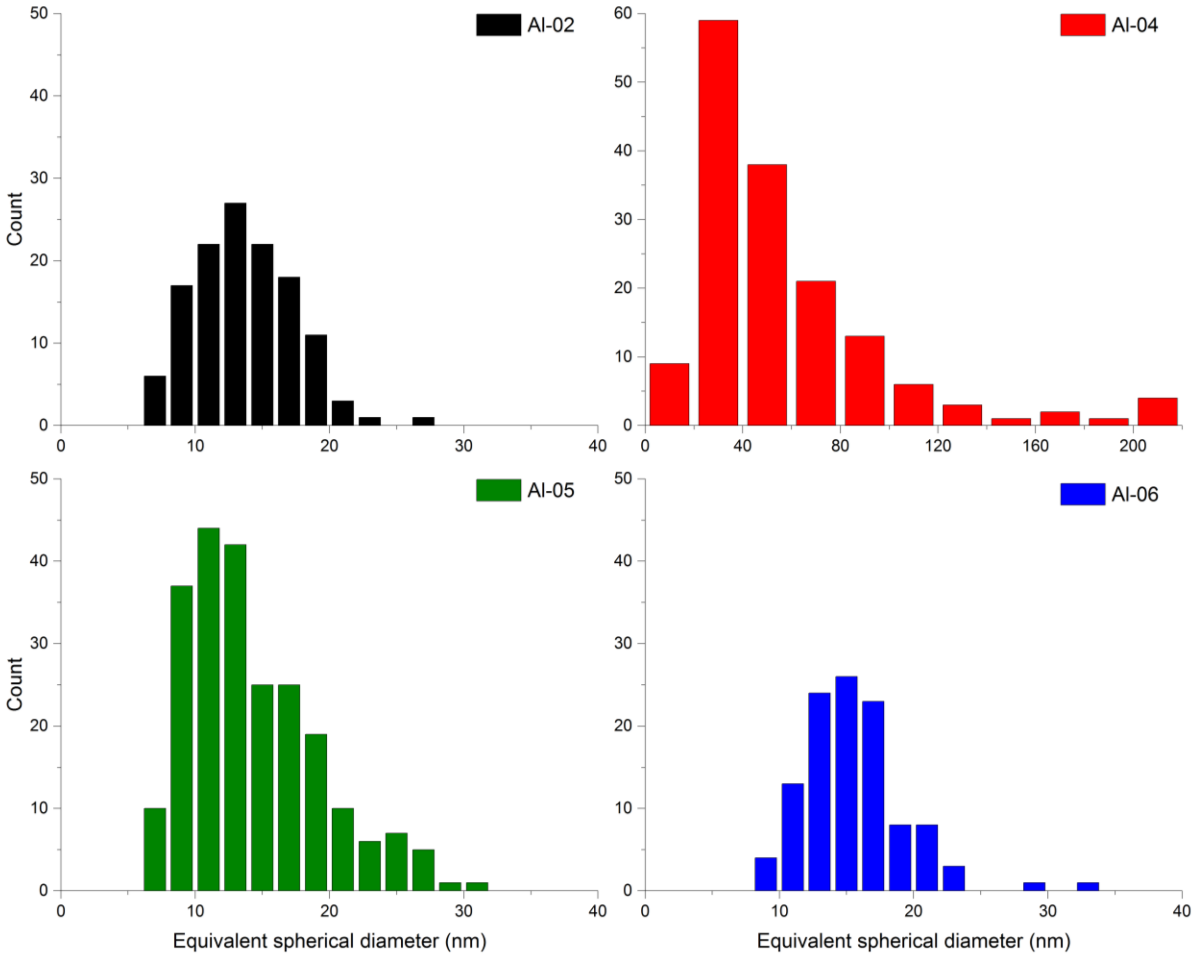


**Figure S35**. Histograms for equivalent spherical diameter for $Al_2O_3$ nanomaterials.

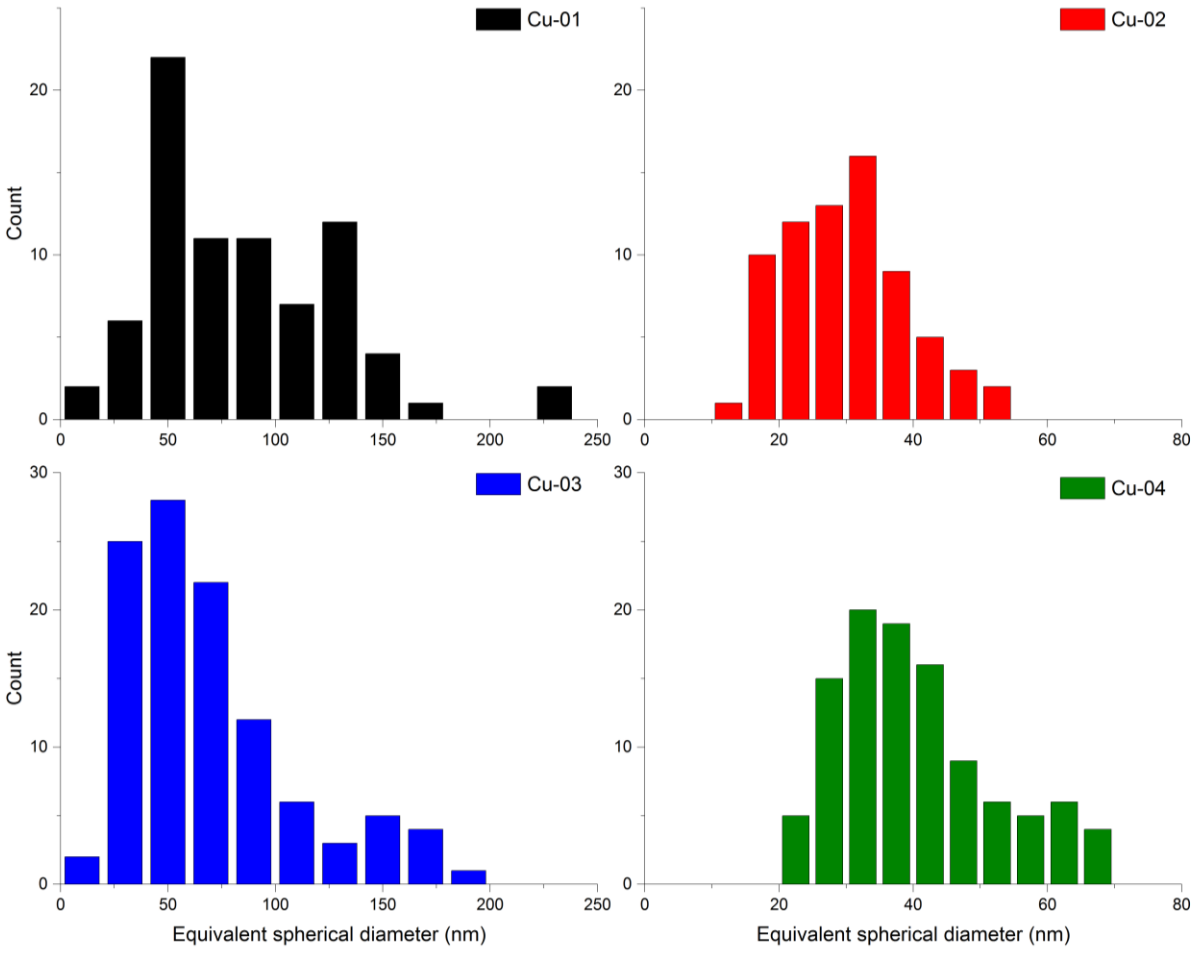


**Figure S36**. Histograms for equivalent spherical diameter for CuO nanomaterials.

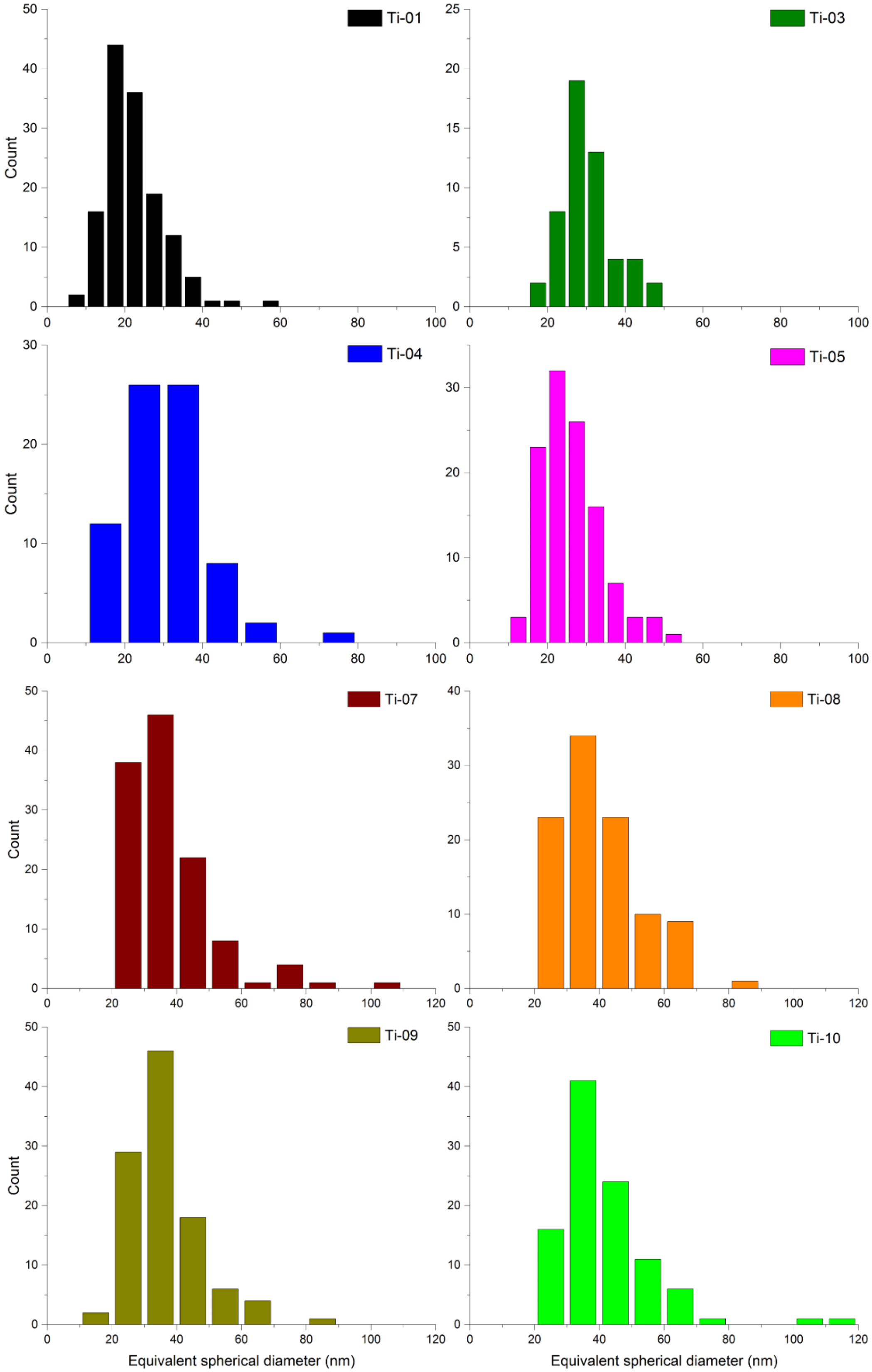


**Figure S37**. Histograms for equivalent spherical diameter for $TiO_2$ nanomaterials.

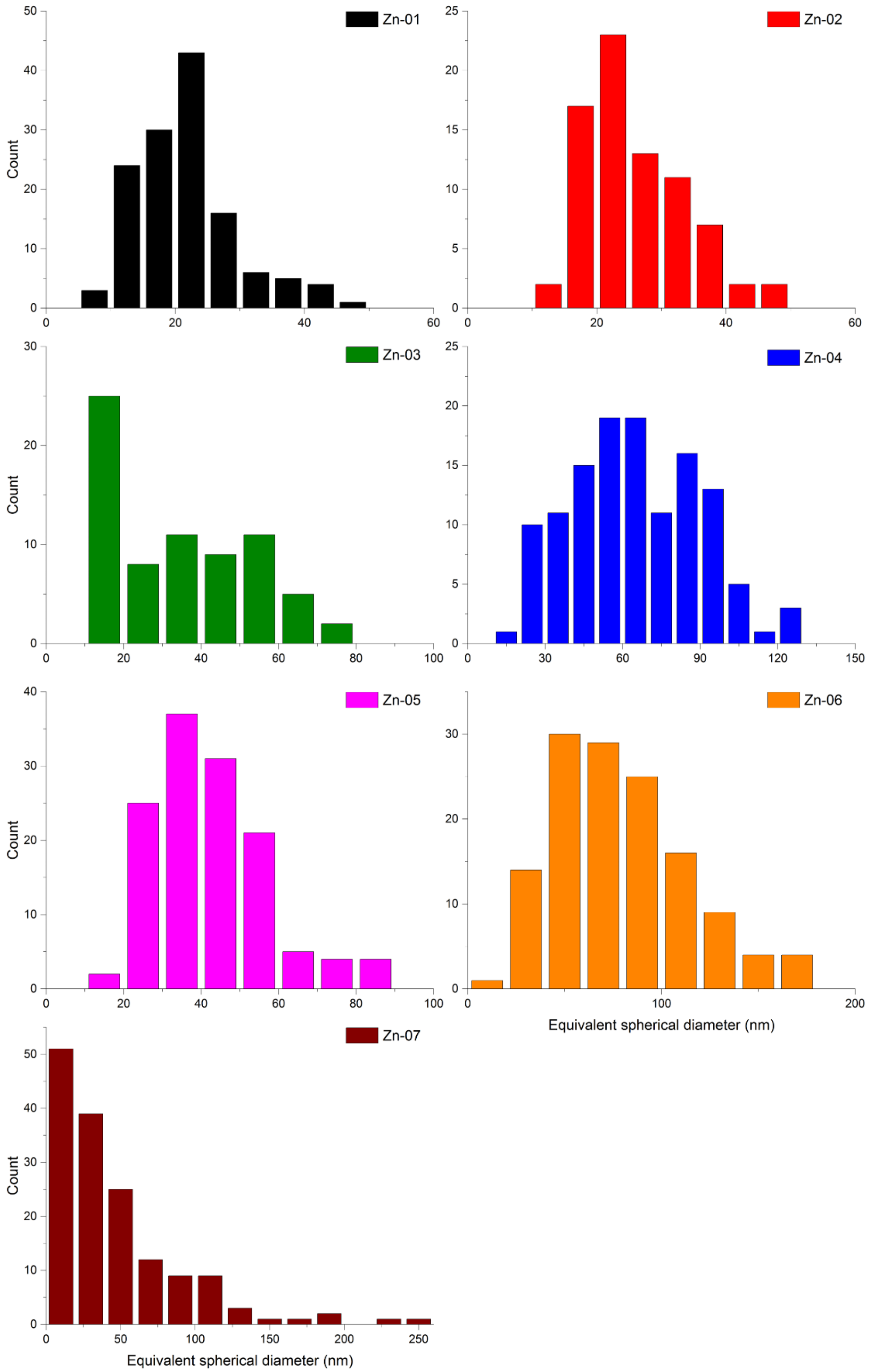


**Figure S38**. Histograms for equivalent spherical diameter for ZnO nanomaterials.
